# Immunoglobulin utilization in Canada: a comparative analysis of provincial guidelines and a scoping review of the literature

**DOI:** 10.1186/s13223-023-00841-z

**Published:** 2023-09-16

**Authors:** Megan Harmon, Kiarash Riazi, Jeannie Callum, Donald M. Arnold, Rebecca Barty, Davinder Sidhu, Nancy M. Heddle, Laurie MacLeod, Na Li

**Affiliations:** 1 Department of Community Health Sciences, Cumming School of Medicine, University of Calgary, CWPH 5E37, 3280 Hospital Dr NW, Calgary, AB T2N 4Z6 Canada; 2 Michael G. DeGroote Centre for Transfusion Research, McMaster University, Hamilton, ON Canada; 3 Ontario Regional Blood Coordinating Network, Hamilton, ON Canada; 4 Department of Pathology and Molecular Medicine, Kingston Health Sciences Centre and Queen’s University, Kingston, ON Canada; 5 Department of Laboratory Medicine and Molecular Diagnostics, Sunnybrook Health Sciences Centre, Toronto, ON Canada; 6 Department of Laboratory Medicine and Pathobiology, University of Toronto, Toronto, ON Canada; 7 Centre for Innovation, Canadian Blood Services, Ottawa, ON Canada; 8 Department of Medicine, Michael G. DeGroote School of Medicine, McMaster University, Hamilton, ON Canada; 9 Cumming School of Medicine, University of Calgary, Calgary, AB, Canada; 10 Department of Computing and Software, McMaster University, Hamilton, ON Canada

**Keywords:** Drug Utilization, Immunoglobulins, Intravenous, Immunoglobulin replacement therapy, IVIG, SCIG, Practice Guidelines, Utilization trend, Treatment switch

## Abstract

**Background:**

Canada has high immunoglobulin (IG) product utilization, raising concerns about appropriate utilization, cost and risk of shortages. Currently, there is no national set of standardized IG guidelines, and considerable variations exist among the existing provincial guidelines. The aims of this study were: (1) to compare the existing Canadian provincial guidelines on the use of IG products to identify their consistencies and differences and (2) to examine the existing research in Canada on IG supply and utilization following the establishment of IG guidelines to understand the scope of research and pinpoint the gaps.

**Methods:**

A comparative analysis accounted for the differences across provincial IG guidelines. We highlighted similarities and differences in recommendations for medical conditions. A scoping review of citations from MEDLINE, PubMed, Scopus and Embase databases was conducted for studies published from January 01, 2014, to April 12, 2023.

**Results:**

While provincial guidelines represented a considerable overlap in the medical conditions delineated and relatively uniform dose calculations, numerous differences were observed, including in recommendation categories, provision of pediatric dosing, and divergent recommendations for identical conditions based on patient demographics. The scoping review identified 29 studies that focused on the use of IG in Canada. The themes of the studies included: IVIG utilization and audits, the switch from IVIG to SCIG, patient satisfaction with IVIG and/or SCIG, the economic impact of self-administered SCIG versus clinically administered IVIG therapy, and the efficacy and cost-effectiveness of alternative medications to IG treatment.

**Conclusion:**

The differences in guidelines across provinces and the factors influencing IVIG/SCIG use, patient satisfaction, and cost savings are highlighted. Future research may focus on clarifying costs and comparative effectiveness, exploring factors influencing guideline adherence, and evaluating the impact of updated guidelines on IG use and patient outcomes.

**Supplementary Information:**

The online version contains supplementary material available at 10.1186/s13223-023-00841-z.

## Introduction

Immunoglobulin (IG) is a plasma product prepared by purifying and pooling antibodies from thousands of healthy individuals [1, 2]. These products are prepared based on the route of administration as intravenous (IVIG) or subcutaneous (SCIG), both of which are available and widely used in Canada [3]. Unlike IVIG which requires administration by a healthcare professional, SCIG can be self-administered at home [4]. IG products are used to treat patients across a broad spectrum of illnesses [5], such as primary and secondary immune deficiencies (PID, SID), immune thrombocytopenia (ITP), and chronic inflammatory demyelinating polyneuropathy (CIDP). Canada is the 2nd highest global per capita consumer of IG products [5]. According to Canadian Blood Services (CBS), half of the patients who use IG products in Canada have no other treatments available for their condition and therefore depend on them to survive [5, 6]. However, due to the high levels of IG use across a range of indications and the shortage of production facilities in Canada, concerns have been raised regarding Canada's long-term ability to ensure the ongoing supply of IG for patients across the country [7].

In the last decade, Canada has experienced an 8% annual growth in the use of IG products [5, 8]. Some of the leading factors that have contributed to the increase in demand include off-label use [9], inappropriate dosing [10–14], and the expansion of indications [15, 16]. The increased demand has made it exceedingly challenging for the Canadian blood product suppliers, the Canadian Blood Services and Hema-Québec (only for Québec), to meet the demand. Only 17% of the plasma required in Canada is produced using donated plasma within the country [5]. Thus, Canada highly depends on suppliers from other countries, mainly the United States, to meet the demands. The high dependency has resulted in towering expenses, with 66% of the total blood expenditure spent to supply these products [5]. To respond to the high demand for IG, the increase in use over time, the supply challenges, and the high costs associated with its use, and to ensure the receipt of IG products, several provincial guidelines for IG use have been developed [17]. However, no Canadian national standardized protocol is currently available, and there are considerable variations among the existing provincial guidelines. Such inconsistencies among the provincial IG guidelines could cause differences in IG utilization and treatment outcome, affecting demand and supply management [18–20].

This study followed two main goals: We first collected and compared the existing provincial guidelines on using IG products in Canada to identify the consistencies and differences in provincial guidelines. Comparing the provincial guidelines can help develop a standardized approach to IVIG use across the country, which can help identify best practices, help improve patient outcomes, reduce variations in care, reduce costs, and support the development of new treatments. The second goal was to examine the existing literature on IG supply and utilization in Canada following the establishment of the IVIG and SCIG guidelines to understand the scope of existing research and pinpoint the gaps. Such studies at the national or local level can support the development of national utilization guidelines by providing valuable data on treatment outcomes, medication safety profiles, regional unique factors that may influence medication responses, cost-effectiveness, and resource availability. This study highlights the need for further research to assess the feasibility of the IVIG and SCIG infusion guidelines and to explore the factors influencing guideline adherence, costs, and comparative effectiveness. It also emphasizes the importance of evaluating the impact of updated guidelines on IG use and patient outcomes to ensure the continued availability of IG products for patients in Canada.

## Methods

### Canadian IG guidelines

Canadian IG guidelines differ by province and have been updated periodically. There are five Provincial guidelines: British Columbia, Ontario, and Québec, a collective guideline for the prairie provinces (Alberta, Saskatchewan, and Manitoba), and one for the Atlantic provinces (New Brunswick, Nova Scotia, Prince Edward Island, and Newfoundland and Labrador). Additional file 1: Table S1 provides overall descriptive details and access links to the guidelines from each province.

To assess the definitions and characteristics within these guidelines, we conducted a comparative analysis, accounting for their differences across provinces and their multiple versions over time. We provided an overview of similarities and differences by summarizing the number of indications for medical conditions, stratified according to recommendation categories, for each guideline. Furthermore, we performed a meta-analysis using random-effects models to assess the consistency of medical recommendations across provincial guidelines. The results are reported in a forest plot, including the proportions of the recommended indications for each medical specialty and their 95% confidence intervals (CIs), the pooled proportions with 95% CIs, and the measure of heterogeneity (I^2^).

### Literature search strategy and selection criteria

We conducted a scoping review to provide a comprehensive overview of the existing publications on IVIG and SCIG utilization in Canada. The review followed the guidelines of the Preferred Reporting Items for Systematic Review and Meta-Analyses extension for Scoping Reviews (PRISMA-ScR) [21]. On April 12, 2023, a literature search was conducted on four databases, MEDLINE and Embase (using the Ovid search platform), PubMed, and Scopus, to search for research papers and conference presentations published since January 2014. The implementation of IVIG guidelines began in Canada in 2012; however, a significant increase in IG product utilization was noted in 2014. The search algorithms for each database are provided in Additional file 1: Table S2.

After the duplicates were removed, the titles and abstracts of the citations were screened by two authors (MH, KR, or NL). Studies were excluded if: they were on animal subjects, emphasizing the biological aspects of the disease and/or treatment, were in languages other than English or French, did not include data from Canada, and were not focused on IG products where "IVIG" or "SCIG" were mentioned only briefly, or if they were unrelated to IG products or transfusion medicine where "IVIG" or "SCIG" had different meanings. For the short-listed citations, full texts were obtained upon availability and examined in duplicates by KR and NL for eligibility assessment to include in the review. Consensus discussions were used to resolve disagreements. Studies that lacked quantitative analysis or were irrelevant to IVIG or SCIG utilization or the research objectives were excluded at this stage.

The information obtained from the selected articles included title, author, publication year, IG product type (IVIG, SCIG, or both), study design, the research question, and the studied variable (e.g., medical condition, adverse effects, utilization). For each study, we provided a summary that included the study cohort, time period, sample size, study design and analysis, and the key findings. We then compared the studies by category to facilitate a thorough understanding of the research landscape.

## Results

### Canadian IG guidelines

Table 1 summarizes the provincial guidelines on IG use in Canada. It includes information such as the version in use, year of release, organization committee, number of indications, availability of a dose calculator, presence of a home infusion program for SCIG, and the materials included in each guideline. The guidelines vary regarding the number of indications covered and the materials included. Except for Quebec, all provincial guidelines include an online dose calculator and a home infusion program for SCIG. Online IVIG dose calculators are available and use the same calculation as shown in the footnote of Table 1. The dose calculators are intended to be used when determining the dose of IVIG for clinically obese patients. The SCIG home infusion program is a valuable support system designed to assist patients undergoing SCIG therapy. It offers in-person training for patients, training partners, and caregivers, streamlines product ordering, and provides continuous case-management services [22]. The program is either administered and funded by provincial health services [22, 23] or pharmaceutical companies [24, 25].

**Table 1:** Summary of provincial guidelines on IG product use in Canada

IG Guidelines	Version in Use (Year)	Organization Committee	Number of Indications	IVIG Dose Calculator^d^	Disclaimer of Dose Calculator	Home Infusion Program	Materials Included
Ontario	V4, 2018	The Ontario Regional Blood Coordinating Network (ORBCoN)	37	Yes	The dose calculator is intended to be used when determining the dose of IVIG for clinically obese patients; it is not recommended for pediatric patients or any patients (including adults) under 5ft in height. The dosing information recommended in the IVIG Utilization Management guidelines should be followed for other patients	Yes	Recommendation categories; Dosing; IG request forms; Infusion guide and adverse reaction chart; Information for outpatients; Documentation for travel
Prairies (Alberta, Saskatchewan, Manitoba)	V2, 2022	The Inter-Provincial Medical Expert Committee and the Institute of Health Economics (IHE)	143	Yes^a^	Same as the Ontario calculator, with the following additional information: Height must be 153–241 cm (60–95 inches). Weight must be 20–400 kg (44–882 pounds)	Yes (AB)	Recommendation categories; Dosing; SCIG administration; Frequency of follow-up and assessment of effectiveness; Instructions for weaning patients off IG, Off-label use; Vaccinations; Adverse effects
Atlantic (New Brunswick, Nova Scotia, Prince Edward Island, and Newfoundland and Labrador)	V2, 2022	The Atlantic Blood Utilization Strategy (ABUS) Working Group	74	Yes^c^	Use the dose calculator if the patient is over 152.4 cm in height and over 50 kg for males/45.5 kg for females	Yes	Recommendation categories; Dosing; SCIG administration
British Columbia	V5, 2019	The BC Provincial Blood Coordinating Office (PBCO)	25	Yes	Separate IVIG dose calculations for adults (18 years old and over), children (under 18 years old), and pregnant women. A dose calculator for SCIG is also available	Yes	Recommendation categories; Dosing
Québec	2017–2022	The Institut national d’excellence en sante et en services sociaux (INESSS)	165	Yes^b^	N/A^b^	No	Recommendation categories; Dosing; Frequency of administration; Adverse reactions; Relative contraindications; Main precautions

IVIG: intravenous immunoglobulin; SCIG: subcutaneous immunoglobulin; IG: immunoglobulin

^a^Prairie guideline: Saskatchewan uses the Alberta dose calculator. Manitoba has its own calculator; however, the same formula is used. (https://healthproviders.sharedhealthmb.ca/services/diagnostic-services/transfusion-manitoba/resources-and-tools/immune-globulin-utilization/)

^b^The Quebec guideline for Infectious Disease, Transplant Medicine, and Rheumatology refers to a dose calculator. However, an online dose calculator is not available. We cannot confirm the equations used for the dose calculation in Quebec

^c^Atlantic guideline: Newfoundland and Labrador have their own calculator; however, the same formula is used (https://www.gov.nl.ca/hcs/bloodservices/resources/dosage-calculator/). There are no specific calculators for New Brunswick and Prince Edward Island

^d^All available IVIG dose calculators use the same equations as follows:

1. The Ideal Boday Weight (IBW) is calculated by: Ideal Body Weight (male) = 50.0 kg + 2.3 kg (each inch > 5 feet or each 2.5 cm > 150 cm); Ideal Body Weight (female) = 45.5 kg + 2.3 kg (each inch > 5 feet or each 2.5 cm > 150 cm)

2. Dosing Weight is an adjusted body weight. It should only be used to calculate the dose of drugs for which there are recommendations specifying that the actual body weight should be adjusted for use in the dose calculations: If Actual Body Weight ≥ IBW, Dosing weight = IBW + 0.4 (Actual Body Weight–IBW); If Actual Body Weight < IBW, Dosing weight = Actual Body Weight

The recommendation categories also vary across the provinces (Table 2). The categories include "recommended"/"approved/recommended"/"do (accepted, effective)"/"indicated conditions" as Level 1, "not recommended for routine use"/"possible treatment option"/"possibly indicated conditions" as Level 2, "not recommended"/"do not do" as Level 3, and "insufficient data"/"do not know" as Level 4 in different provincial guidelines. In Table 3, we reported the number of indications in each medical specialty (dermatology, hematology, immunology, infectious disease, transplant medicine, neurology, and rheumatology) for each province and the percentage of indications that fall into each recommendation category. Overall, most indications for IG use in Ontario, Prairies, Atlantic, and Québec fall into the two categories with high uncertainty, Level 2 or Level 4, accounting for 43%, 38%, 59%, and 55%, respectively. British Columbia has the highest percentage of Level 1 "recommended" at 64% due to their two-level recommendation strategy, which includes only Level 1 and Level 3.

**Table 2:** Provincial IG guideline recommendation categories

Category level	Ontario	Prairies	Atlantic	British Columbia	Québec
1	Recommended	Do (accepted, effective)	Indicated conditions	Approved/recommended	Recommended
2	Not recommended for routine use		Possibly indicated conditions		Possible treatment option
3		Do not do (evidence does not support)		Not recommended	Not recommended
4		Do not know (insufficient evidence)			Insufficient data

**Table 3:** Guideline comparison summary table

Medical condition category	Number of indications				
	Ontario	Prairie	Atlantic	British columbia	Québec
Dermatology	N = 2^a^	N = 16	N = 16	N = 1	N = 15
	Recommended: 1 (50%) Not recommended for routine use: 1 (50%)	Do: 4 (25%) Do not do: 0 Do not know: 12 (75%)	Indicated conditions: 4(25%) Possibly indicated conditions: 12(75%)	Approved/Recommended: 1 (100%) Not recommended: 0	Recommended: 2 (13%) Possible option: 7 (47%) Not recommended: 4 (27%) Insufficient data: 2 (13%)
Hematology	N = 14	N = 22	N = 16	N = 5	N = 23
	Recommended: 5 (36%) Not recommended for routine use: 9 (64%)	Do: 12 (55%) Do not do: 3 (14%) Do not know: 7 (32%)	Indicated conditions: 9 (56%) Possibly indicated conditions: 7 (44%)	Approved/Recommended: 4 (80%) Not recommended: 1 (20%)	Recommended: 5 (22%) Possible option: 9 (39%) Not recommended: 7 (30%) Insufficient data: 2 (9%)
Immunology	N = 2^a^	N = 2	N = 5	N = 2	N = 42
	Recommended: 2 (100%) Not recommended for routine use: 0	Do: 2 (100%) Do not do: 0 Do not know: 0	Indicated conditions: 4 (80%) Possibly indicated conditions: 1 (20%)	Approved/Recommended: 2 (100%) Not recommended: 0	Recommended: 11 (26%) Possible option: 11 (26%) Not recommended: 11 (26%) Insufficient data: 9 (21%)
Infectious Disease​	N = 2	N = 11	N = 4	N = 3	N = 13
	Recommended: 2 (100%) Not recommended for routine use: 0	Do: 4 (36%) Do not do: 6 (55%) Do not know: 1 (9%)	Indicated conditions: 2 (50%) Possibly indicated conditions: 2 (50%)	Approved/Recommended: 3 (100%) Not recommended: 0	Recommended: 0 Possible option: 3 (23%) Not recommended: 7 (54%) Insufficient data: 3 (23%)
Transplant Medicine​	N = 4	N = 21	N = 3	N = 0	N = 11
	Recommended: 4 (100%) Not recommended for routine use: 0	Do: 6 (28%) Do not do: 9 (43%) Do not know: 6 (28%)	Indicated conditions: 1 (33%) Possibly indicated conditions: 2 (67%)		Recommended: 0 Possible option: 5 (45%) Not recommended: 2 (18%) Insufficient data: 4 (36%)
Neurology	N = 10	N = 49	N = 19	N = 12	N = 27
	Recommended: 4 (40%) Not recommended for routine use: 6 (60%)	Do: 15 (31%) Do not do: 16 (32%) Do not know: 18 (37%)	Indicated conditions: 6 (32%) Possibly indicated conditions: 13 (68%)	Approved/Recommended: 4 (33%) Not recommended: 8 (67%)	Recommended: 4 (15%) Possible option: 9 (33%) Not recommended: 9 (33%) Insufficient data: 5 (19%)
Rheumatology	N = 3	N = 22	N = 11	N = 2	N = 34
	Recommended: 3 (100%) Not recommended for routine use: 0	Do: 8 (36%) Do not do: 4 (19%) Do not know: 10 (45%)	Indicated conditions: 4 (36%) Possibly indicated conditions: 7 (64%)	Approved/Recommended: 2 (100%) Not recommended: 0	Recommended: 1 (3%) Possible option: 12 (35%) Not recommended: 11 (32%) Insufficient data: 10 (29%)
**Total**	**N = 37**	**N = 143**	**N = 74**	**N = 25**	**N = 165**
	Level 1: Total recommended: 21 (57%) Level 2: Total not recommended for routine use: 16 (43%)	Level 1: Total do: 51 (36%) Level 3: Total do not do: 38 (26%) Level 4: Total do not know: 54 (38%)	Level 1: Total indicated conditions: 30 (41%) Level 2: Total possibly indicated conditions: 44 (59%)	Level 1: Total Approved/recommended: 16 (64%) Level 3: Total not recommended: 9 (36%)	Level 1: Total recommended: 23 (14%) Level 2: Total possible option: 56 (34%) Level 3: Total not recommended: 51 (31%) Level 4: Total insufficient data: 35 (21%)

The specific medical conditions under each category can be found in Additional file 1: Table S5

^a^Medical conditions in the Ontario guideline considered further grouping where toxic epidermal necrolysis (TEN) and Stevens-Johnson syndrome (SJS) were grouped in dermatology, and primary immune deficiency (PID) and secondary immune deficiency (SID) were grouped in immunology

**Fig. 1:**
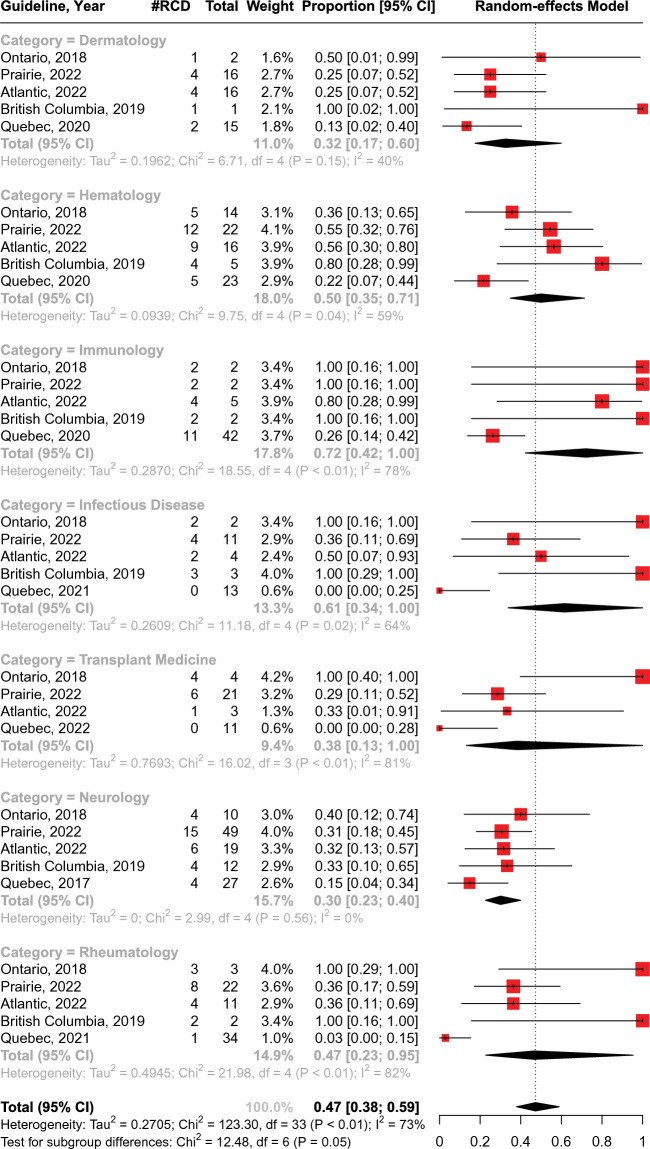
Forest plot for the proportions of the "recommended" medical indications under each medical specialty per provincial guidelines. #RCD: Number of "recommended" medical indications. Each red square represents the proportion of medical indications that fall in each provincial guideline's "recommended" category. The extending lines represent the 95% confidence intervals of the proportions. The black diamonds represent the pooled proportions calculated from the random-effects models for each medical specialty and all specialties

Figure 1 displays a forest plot from a meta-analysis that evaluates the recommendations for IVIG use across provincial guidelines, stratified by medical specialties. When ranked by the percentage of indications for which IVIG was recommended, the order from highest to lowest was immunology (72%), infectious disease (61%), hematology (50%), rheumatology (47%), transplant medicine (38%), dermatology (32%), and neurology (30%). However, significant heterogeneity measured by I^2^ was observed in the guidelines within the medical specialties. Among the 7 specialties, 5 specialties had an I^2^ greater than 50%: rheumatology (82%), transplant medicine (81%), immunology (78%), infectious disease (64%), and hematology (59%). Across all medical indications for all guidelines, the pooled percentage of the "recommended" indications was 47% (95% CI 38%, 59%), with a high overall heterogeneity of I^2^ = 73%.

### Scoping review of current literature

**Fig. 2:**
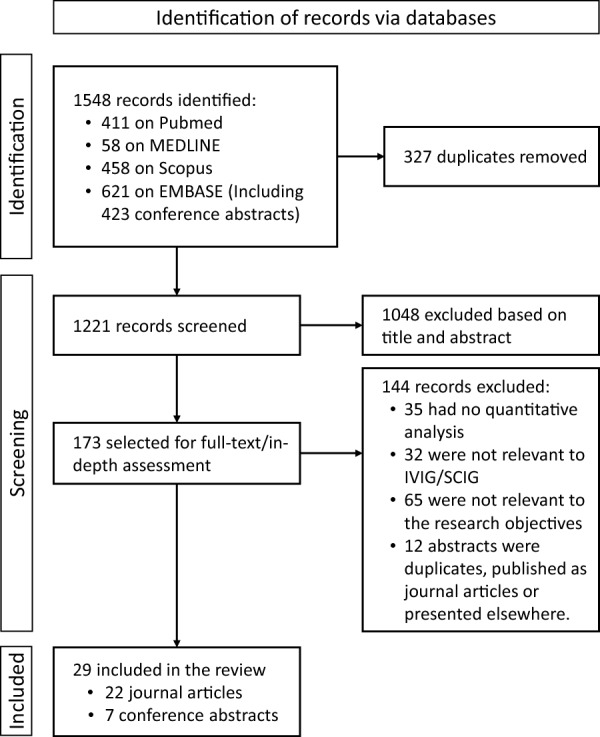
PRISMA flow diagram for scoping review study selection

Figure 2 represents the study selection process. Our citation search yielded 1548 articles, of which 29 were eligible for review, including 22 journal articles and 7 conference abstracts (Table 4).

**Table 4:** Summary of included studies

Study	Type of work	Location	Sample Size / Cohort	IG type	Study period	Study Design/Analysis
Alcantara et al. (2021) [32]	Journal article	Single site in Toronto, Ontario	34 patients with generalized MG	IVIG & SCIG	2015–2020	Retrospective cohort study
Arnold et al. (2020) [50]	Journal article	Canada-wide	74 patients with ITP	IVIG vs eltrombopag	2013–2019	Clinical trial (randomized, open-label)(Bridging ITP Trial)
Bourque et al. (2016) [31]	Journal article	Single site in Ottawa, Ontario	9 patients with MG	IVIG & SCIG	2015–2016	Retrospective cohort study
Brownlee et al. (2022) [48]	Journal article	Single site in Ottawa, Montreal	10 patients with PID or SID who did not tolerate the side effects of 20% SCIG	SCIG	2018–2020	Prospective cohort
Fu et al. (2018) [35]^a^	Journal article	Single site in Toronto, Ontario	57 PID patients	SCIG & IVIG	Study published in 2018-	Cost savings study
Gerth et al. (2014) [34]^a^	Journal article	N/A	PID/SID patients	IVIG & SCIG	N/A	Cost savings simulation study
Hsia et al. (2015) [27]	Journal article	Two hospitals in Ontario	383 adult patients with ITP	IVIG	2003–2012	Retrospective audit
Jutras et al. (2021) [26]	Journal article	Single site in Montréal Québec	172 critically ill pediatric patients	IVIG	2013–2018	Retrospective cohort study
Kaur et al. (2022) [51]	Journal article	Canada-wide	74 patients with ITP	IVIG vs eltrombopag	2013–2019	Cost-effectiveness analysis of data from Bridging ITP Trial
Keith et al. (2022) [46]^a^	Journal article	6 centers across Canada (except Québec)	125 Patients with PID or SID	SCIG	2018–2020	Phase 4, non-interventional, prospective, single-arm study
Kobayashi et al. (2022) [47]^a^	Journal article	Multi-national, including Canada	102 patients with PID	SCIG	238 weeks follow-up duration. Dates were not mentioned	Secondary analysis of data from previous single-arm phase 3 trials
Liu et al. (2019) [28]	Journal article	Single site in Toronto, Ontario	40 patients with ITP	IVIG	2014	Audit
Mallick et al. (2022) [41]^a^	Journal article	Canada-wide	296 Patients with PID or SID	SCIG & IVIG	2020–2021	Survey Study
Murphy et al. (2019) [17]	Journal article	Four sites in Ottawa, Ontario	2629 patients	IVIG	2007–2016	Trend Analysis
Reid et al. (2014) [37]	Journal article	Referral centers or community hospitals in Ontario	169 patients who were on hospital-based IG replacement therapy	IVIG & SCIG	–	Survey using questionnaire
Ritchie et al. (2022) [36]^a^	Journal article	Alberta, Province-wide	7890 adult and pediatric patients who used IG products	SCIG & IVIG	2012–2019	Retrospective population-based cohort
Shih et al. (2017) [9]	Journal article	Four sites in Ontario	178 adult patients	IVIG	2014	Audit
Sholapur et al. (2016) [38]	Journal article	Single site in Ontario	12 ITP patients	IVIG	Study published in 2016-	Cross-sectional study
Suleman et al. (2019) [33]^a^	Journal article	Single site in Ottawa, Ontario	19 patients with immune-mediated neurological disorders	IVIG & SCIG	2010–2016	Retrospective cohort study
Sultan et al. (2017) [39]^a^	Journal article	Single site in Montreal, Québec	60 children with PID	SCIG	Study published in 2017-	Cross-sectional study
Tran et al. (2023) [29]	Journal article	Three hospitals in British Columbia	114 patients with SID	SCIG & IVIG	2018–2019	Retrospective cohort/audit
Walter et al (2014) [45]	Journal article	Manitoba, province-wide	62 patients with PID	SCIG	2007–2018	Retrospective
Abadeh et al. (2023) [49]	Conference abstract	Ontario, multicentre	108 patients with SID	IVIG/SCIG	2020–2022	Cross-sectional
Furlan et al. (2016) [52]	Conference abstract	Ontario	32 patients who received IVIG and 38 patients treated with plasma exchange	IVIG	2007–2010	Cost-minimization analysis
Mallon et al. (2016) [43]	Conference abstract	Alberta, multicentre	8 patients with MG	SCIG	–	Clinical trial
Shabani-Rad et al. (2018) [30]	Conference abstract	Calgary, Alberta	IVIG recipients	IVIG	2017	Cross-sectional
Siddiqi et al. (2018) [44]	Conference abstract	Alberta, multicentre	822 patients with MG	SCIG	–	Clinical trial
Streu et al (2016) [42]	Conference abstract	Manitoba, single centre	53 patients with CLL	SCIG	Two year period	Prospective
Zhou et al. (2021) [40]	Conference abstract	Ontario, single centre	Adult patients receiving IVIG for Inflammatory Myositis	IVIG/SCIG	–	Survey

IVIG: intravenous immunoglobulin; SCIG: subcutaneous immunoglobulin; MG: myasthenia gravis; ITP: immune thrombocytopenia; PID: primary immune deficiency; SID: secondary immune deficiency. The key findings of the publications in this table can be found in Additional file 1: Table S3

^a^Studies were funded by IVIG/SCIG manufacturers. Fu et al., Gerth et al., Mallick et al., Ritchie et al., Suleman et al., and Sultan et al. were funded by CSL Behring. Keith et al. were funded by Baxalta US Inc. and Baxalta Innovations GmBH. Kobayashi et al. was funded by Octapharma AG

#### IVIG utilization and trend

Three studies investigated IVIG utilization, though in different contexts. Murphy et al. [17] focused on trends in IVIG use and the impact of provincial use mitigation strategies in a tertiary care center, Jutras et al. [26] investigated IVIG indications in a pediatric intensive care unit, and Hsia et al. [27] assessed the utilization of IVIG in patients with ITP and attempted to forecast future demand. These studies highlighted the rising trend in IVIG use. Murphy et al. [19] also revealed a slowed increase after implementing provincial use mitigation strategies. Both studies by Murphy and Jutras emphasized the limitations in the existing interventions and guidelines to control IVIG use, with the latter explicitly noting a high prevalence of off-label IVIG administration [26]. These findings suggest the need for reinforcement strategies, a better understanding of guideline adherence factors, and an exploration of alternative explanations for changes in IVIG use.

#### Audit of IVIG use

Shih et al. [9] and Liu et al. [28] examined IVIG use in different settings in Ontario. Shih et al. conducted a retrospective multicentre audit to understand the factors associated with increased IVIG use, while Liu et al. performed a hospital chart review of patients receiving IVIG for ITP. Both studies identified challenges in ensuring appropriate IVIG use. Shih et al. found low compliance with the Ontario IVIG Utilization Management Strategy and deficiencies in the completion of the IVIG Request Form, while Liu et al. observed that the use of IVIG for ITP was generally appropriate and carefully evaluated even in cases where the current provincial recommendations were not met. The studies concluded with recommendations for improving IVIG use, including a comprehensive, evidence-based active surveillance process and the development of clinical guidelines for IVIG use to ensure an appropriate and cost-effective treatment [9, 28].

More recently, Tran et al. [29] audited IG replacement therapy for SID at three hospitals in British Columbia. They evaluated the appropriateness of IG treatments against the Australian BloodSTAR Guidelines as a robust benchmark and showed that almost half (48%) of the study population had inappropriate IG replacement therapy. The most common reason was a lack of proper follow-up IgG levels at 6 or 12 months. Their data indicated a need for stringent guidelines to guide ordering practices [29]. Also, Shabani-Rad et al. [30] have developed and recommended a structured IVIG utilization program and comprehensive database in Alberta to manage and monitor IVIG utilization effectively. In collaboration with clinical disciplines, their review of registered patients labelled 85% of cases as appropriate.

#### Chronic IG therapy: from IVIG to SCIG

Bourque et al. [31], Alcantara et al. [32], and Suleman et al. [33] conducted retrospective cohort studies examining the use of SCIG in the treatment of myasthenia gravis (MG) and immune-mediated neurological disorders. Bourque et al. explored the use of SCIG in chronic inpatient MG management, observing a stable or improved MGFA (Myasthenia Gravis Foundation of America) clinical classification after SCIG initiation, with significant improvements in Myasthenia Gravis Activities of Daily Living (MG-ADL) profile and Myasthenia Gravis Quality-of-life (MG-QOL) [31]. Alcantara et al. studied the long-term effects of chronic immunoglobulin maintenance therapy (IVIG and/or SCIG) in MG treatment in-hospital, finding significant reductions in the number of immunosuppressive medications, prednisone and pyridostigmine doses with chronic IG treatment [32]. Finally, Suleman et al. examined the SCIG home infusion program for immune-mediated neurological disorders and reported high success rates in transitioning patients from IVIG to SCIG [33].

#### Cost saving analysis: from IVIG to SCIG

Two papers investigated the potential economic benefits of switching patients with PID/SID from IVIG to home-based SCIG therapy. Gerth et al. [34] used simulation modelling to estimate the impact on nursing time and cost savings. Switching a significant portion of patients from IVIG to SCIG could save 223.3 nurse FTEs (CAD$ 23.2 million in labour costs), potentially alleviating nurse shortages in Canada. Fu et al. [35] compared hospital and physician costs for patients receiving SCIG versus IVIG and found significantly lower average total costs for the SCIG group from the hospital's and physician's perspectives than the IVIG group.

Similarly, a comparative cost analysis by Ritchie et al. [36] between SCIG administration at home and IVIG in clinical settings showed that the self-administration of SCIG would save an average of $5386 per patient annually. Their results indicated that if 50% of patients who only received clinic-administered IVIG switched to self-administered SCIG during their study period, that would have saved $19.4 million for the Canadian healthcare system [36].

#### Patient satisfaction with IG therapy

A survey study by Reid and Pires [37] on the experiences of patients and their preferences for IG treatment and their willingness to switch to a home-based program offered the home-based program as a potentially preferred option for patients with immune deficiency. Although patients mostly preferred IVIG over SCIG then, the loss of time and travel costs associated with hospital-based programs were considerable factors favouring home-based programs. Later on, Sholapur et al. [38] and Sultan et al. [39] investigated patient satisfaction with IVIG and SCIG treatments, respectively. Sholapur et al. evaluated IVIG's effectiveness and patient satisfaction as a treatment for ITP and found IVIG treatment to be perceived as inconvenient but satisfactory in terms of tolerability for ITP management [38]. Sultan et al. assessed the quality of life, treatment beliefs, and satisfaction among children with PID treated with SCIG. They reported that SCIG treatment was well-received by children and improved their quality of life. However, they also highlighted potential adverse effects, such as injection site reactions, which could affect treatment satisfaction [39].

In a recent report, Zhou et al. [40] studied patient satisfaction with IVIG treatment and their perceptions of SCIG for Inflammatory Myositis. They showed that while most patients were satisfied with the effectiveness of IVIG treatment, many found it inconvenient mainly due to its side effects. However, their willingness to switch to SCIG was low, possibly due to unfamiliarity with the treatment. Mallick et al. [41] surveyed the treatment satisfaction of adults receiving IVIG or SCIG for PID or SID. The patients receiving SCIG had spent significantly less infusion preparation time, actual infusion time, and post-infusion clean-up time per infusion than IVIG users. The SCIG recipients reported better treatment satisfaction than IVIG patients regarding perceived effectiveness. Patients who transitioned from IVIG to SCIG were overall satisfied with the experience, with many respondents reporting improved health-related quality of life, productivity, physical and mental health, and greater treatment satisfaction and compliance.

#### Efficacy and safety of SCIG

Several studies evaluated the efficacy and safety of SCIG treatment. The study by Streu et al. [42] confirmed that SCIG treatment in patients with chronic lymphocytic leukemia (CLL) was not only effective but also resulted in significant cost savings, improved quality of life and treatment satisfaction. Similarly, in patients with MG exacerbation, a multicenter clinical trial in Alberta on the efficacy, safety and feasibility of 20% SCIG (Hizentra) indicated that SCIG was effective in treating mild to moderate exacerbations [43, 44]. Despite its large volume, SCIG appeared well tolerated at the standard IVIg dose with mild and rare local or systemic side effects [43].

Other studies focused on immune deficiencies in general. Walter et al. [45] showed that SCIG push (the administration of SCIG using butterfly needles and a syringe) to adults with PID was both effective (It significantly increased the serum IgG levels and effectively prevented infections compared to before treatment started) and well accepted by patients. A recent study by Keith et al. [46] evaluated the safety and patient satisfaction with 20% SCIG solution (Ig20Gly) in PID or SID patients after switching from other SCIG products. The patients under treatment with Ig20Gly maintained protective IgG levels against infections at 6 and 12 months after treatment initiation. Most adverse effects were generally mild to moderate and mainly included headaches or infusion-site reactions, and all patients expressed an interest in continuing Ig20Gly treatment [46]. Similarly, the research by Kobayashi et al. [47] suggested that Cutaquig (a 16.5% SCIG preparation) maintained efficacy and had mild and transient local and systemic adverse reaction rates in PID patients over four years of follow-up. Additionally, Brownlee et al. [48] also showed that Cutaquig could be an alternative treatment option for patients who could not tolerate the side effects of 20% SCIG products. They also reported no serious or severe adverse events while providing therapeutic levels of serum IgG in immunodeficient patients. Nonetheless, both IVIG and SCIG treatments in patients with SID are effective by reducing the number of infections and emergency department visits and improving patient perception of health compared to before treatment, as demonstrated by Abadeh et al. [49].

#### Alternative therapies for IVIG

Considering the cost and supply challenges related to IVIG and the associated side effects, including headache, aseptic meningitis, and allergic reactions, alternative treatment options have been considered for some of their medical indications. We found one study that compared the efficacy and safety of eltrombopag (a thrombopoietin receptor agonist indicated for chronic ITP) with those of IVIG. IVIG is commonly used to increase the platelet count before surgery for patients with ITP because it can induce a rapid and transient rise in the platelet count [50]. A study by Arnold et al. [50] showed that eltrombopag was non-inferior to IVIG for achieving and maintaining platelet count during the 7 day perioperative period. However, rare cases of serious side effects (pulmonary embolism and rebound thrombocytopenia) in the eltrombopag group suggested that the medication could be used as an alternative to IVIG for perioperative management of ITP, but with recommended attention to the risk of thrombosis and platelet count fluctuations. A follow-up study of patient-level data from the same trial was performed by Kaur et al. [51] to analyze the cost-effectiveness of the treatment from a Canadian public healthcare payer's perspective. The cost-effectiveness analysis indicated that eltrombopag was both more effective and less costly than IVIG, and perioperative eltrombopag saved an average of $413 Canadian per patient over the study period. Yet, a cost-minimization analysis by Furlan et al. [52] to compare IVIG with plasma exchange, two equally effective alternatives for treating patients with MG exacerbation, demonstrated that differences in cost-minimizing for treatments depend on different stakeholders' perspectives.

## Discussion

The scoping review included 29 studies which focused on IVIG utilization and audits, the switch from IVIG to SCIG, patient satisfaction with immunoglobulin therapies, and alternative therapies for IVIG. The review also provided a comprehensive analysis of Canadian IG guidelines across provinces.

We recognized two primary similarities by comparing Canadian IG guidelines across provinces. Firstly, the medical conditions delineated in the IG guidelines exhibited considerable overlap, with minor deviations due to disparities in recommendation categories. Secondly, dose calculations demonstrated relative uniformity among the guidelines. However, numerous differences exist between the provincial IG guidelines. The Atlantic guideline stands out as the only one offering a comprehensive list of dosing recommendations for relevant medical conditions. In contrast, the Prairie and Québec guidelines mention SCIG administration but lack information on dosing. Recommendation categories also exhibit high variations between guidelines, with a high percentage of indications for IG use falling into the categories of "possible treatment option" or "insufficient data" in Ontario, Prairies, Atlantic, and Québec. In contrast, British Columbia distinguishes between recommended and not recommended categories. Moreover, some guidelines group multiple conditions within a single category, while others list them separately (e.g., the Québec guideline enumerates conditions that fall under the PID or SID categories, whereas other guidelines use the general terms 'PID' and 'SID' instead). Other discrepancies include the inconsistent provision of pediatric dosing and divergent recommendations for identical conditions based on patient demographics. The meta-analysis reveals that approximately 50% of the medical indications were recommended for IVIG, with the remaining 50% being not recommended or having an ambiguous recommendation. The high overall heterogeneity (I^2^ = 73%) underscores a significant inconsistency across different provincial guidelines. Furthermore, the proportion of indications recommended for IVIG varied among different medical specialties, ranging from 30% in neurology to 72% in immunology. Substantial heterogeneity was also observed within specialties across the guidelines. These variations in guidelines and recommendations may contribute to inconsistencies in IG use and adherence across the country. It is worth noting that the National Advisory Committee on Blood and Blood Products [53] has issued nationwide recommendations for the administration of fibrinogen [54] and prothrombin complex concentrates [55]. However, standardized national recommendations for the use of IVIG and SCIG are yet to be established.

The findings on IVIG utilization not only revealed an increasing trend in IVIG use but also highlighted the limitations in current interventions and guidelines to control its use [17, 26, 27]. This suggests the need for reinforcement strategies, a better understanding of guideline adherence factors, and exploring other explanations for changes in IVIG use. The audit studies identified challenges in ensuring appropriate IVIG use, with insufficient documentation and low compliance with the Ontario IVIG Utilization Management Strategy [9, 28] or high rates of inappropriate treatments when measured against a robust benchmark [29]. These results call for a comprehensive, evidence-based active surveillance process and the development of clinical guidelines to ensure appropriate and cost-effective treatment.

The studies on chronic IG therapy demonstrated the potential benefits of SCIG, including improvements in disease outcomes, reductions in the use of other medications, and fewer side effects compared to IVIG [31–33]. However, they also suggested areas for further research, such as the need for prospective randomized studies to clarify costs and comparative effectiveness [32] or longer follow-up periods and specific IVIG to SCIG conversion ratios for certain patient populations [33].

The cost-saving analysis studies showed the potential for reduced healthcare resource utilization when switching patients from IVIG to home-based SCIG therapy [34–36]. Furthermore, the availability of several SCIG home infusion programs, entirely funded by pharmaceutical companies [24, 25], contributes to the advantages of home-based SCIG treatments concerning healthcare expenses. These findings help justify the provision of home-based therapy training to suitable patients and can encourage healthcare professionals to consider advocating for home-based SCIG therapy for PID/SID patients when clinically appropriate. The patient satisfaction studies [38, 39, 41] provided insights into patient satisfaction regarding IVIG and SCIG treatments. IVIG was found to be satisfactory in tolerability but inconvenient, while SCIG was generally well-received with improved quality of life and lower risk for systemic adverse effects [56–58]. The studies that compared the efficacy and cost-benefits of alternative treatments to IG products can provide alternative reasonable treatment options, especially in light of IG product supply limitations [50, 51]. This information could be valuable in guiding healthcare providers and policymakers to tailor IG treatment strategies to better suit patients' needs and preferences.

Nevertheless, the existing studies have several limitations: (1) Several studies were conducted at single sites and might not be representative of other regions in Canada. (2) Most studies have small sample sizes, which could limit the generalizability of their results to larger populations. (3) The focus on specific medical conditions, such as ITP, PID, MG, and immune-mediated neurological disorders, means that the results may not be applicable to other medical conditions. (4) Some of the studies are conducted over a relatively short period, which could limit the ability to draw long-term conclusions. (5) Studies with retrospective design can be subject to selection and recall bias (e.g., underreporting of adverse events, unclear principal diagnosis for IG use, reliance on chart reviews for symptom assessment).

This scoping review also has some limitations. The primary focus of our research was to examine the research on IG utilization in Canada, along with its corresponding provincial guidelines. Although other countries might face similar ongoing issues regarding IG supply, it is crucial to recognize that the applicability of our findings and conclusions to different countries or healthcare systems could be restricted. Moreover, the review included a relatively small number of studies, which may not fully reflect the Canadian context. The review focused solely on published literature and conference abstracts, which did not consider gray literature such as unpublished reports or government documents, which may contain important information not published in peer-reviewed journals. Furthermore, the time frame of 2014 to 2023 may result in the omission of relevant papers published before 2014, such as the study by Chow et al. [59]. However, the utilization of IG products was limited before that time.

In conclusion, this study highlights the differences in Canadian IG guidelines across provinces and the various factors influencing IVIG and SCIG use, patient satisfaction, cost savings, and alternative therapies for IVIG. The findings of this review may inform healthcare professionals, policymakers, and guideline developers in their efforts to unify and optimize immunoglobulin therapy practices in Canada. Future research may focus on conducting prospective randomized studies to clarify costs and comparative effectiveness, exploring factors influencing guideline adherence, evaluating the impact of updated guidelines on IG use and patient outcomes, and establishing a unified national IG guideline.

### Supplementary Information

**Additional file 1: Table S1.** Provincial guidelines on the use of IG products in Canada. **Table S2.** Scoping review search terms. **Table S3:** The key findings of the selected publications. **Table S4.** Preferred reporting items for systematic reviews and meta-analyses extension for scoping reviews (PRISMA-ScR) checklist. **Table S5.** Summary of medical conditions by specialty in provincial guidelines.

## Data Availability

Not applicable.

## References

[1] Arumugham VB, Rayi A. Intravenous Immunoglobulin (IVIG). StatPearls. Treasure Island (FL): StatPearls Publishing Copyright ^©^ 2022, StatPearls Publishing LLC.; 2022. https://www.ncbi.nlm.nih.gov/books/NBK554446/]. 32119333

[2] Perez EE, Orange JS, Bonilla F, Chinen J, Chinn IK, Dorsey M (2017). Update on the use of immunoglobulin in human disease: a review of evidence. J Allergy Clin Immunol.

[3] Harding SR, Lazarus A. Chapter 4: Immune globulin products. In: Khandelwal A, Abe T, editors. Canadian Blood Services' Clinical Guide to Transfusion online edition 2018. https://professionaleducation.blood.ca/en/transfusion/clinical-guide/immune-globulin-products (Accessed 01 Feb 2023).

[4] Home-Based Subcutaneous Infusion of Immunoglobulin for Primary and Secondary Immunodeficiencies: A Health Technology Assessment. Ontario health technology assessment series. 2017; 17(16): 1–86. PMC6548531 31210833

[5] Expert Panel on immune globulin product supply and related impacts in Canada. Protecting Access to Immune Globulins for Canadians. Government of Canada Publications, 2018. https://publications.gc.ca/collections/collection_2018/sc-hc/H22-4-12-2018-eng.pdf (Accessed 01 Feb 2023).

[6] Cortes K, Smith L, editors. Pharmaceutical access and use during the pandemic. Statistics Canada; 2022. https://www150.statcan.gc.ca/n1/en/pub/75-006-x/2022001/article/00011-eng.pdf?st=LjjdGSei (Accessed 05 June 2023).

[7] Canadian Blood Services; National Advisory Committee on Blood and Blood Products. The National Plan for Management of Shortages of Immunoglobulin Products (Ig)–Interim Guidance, 2020 [Available from: https://nacblood.ca/sites/default/files/2021-09/The%20National%20Plan%20for%20Management%20of%20Shortages%20of%20Immunoglobulin%20Products%20%28Ig%29%20%20Interim%20Guidance_July%2027%202020.Published.pdf (Accessed 12 Aug 2023).

[8] Office of the Auditor General of Ontario. Value-for-Money Audit: Blood Management and Safety (2020). 2020 https://www.auditor.on.ca/en/content/annualreports/arreports/en20/20VFM_02bloodmgmt.pdf (Accessed 05 June 2023).

[9] Shih AW, Jamula E, Diep C, Lin Y, Armali C, Heddle NM (2017). Audit of provincial IVIG request forms and efficacy documentation in four ontario tertiary care centres. Transfus Med.

[10] Abolhassani H, Sadaghiani MS, Aghamohammadi A, Ochs HD, Rezaei N (2012). Home-based subcutaneous immunoglobulin versus hospital-based intravenous immunoglobulin in treatment of primary antibody deficiencies: systematic review and meta analysis. J Clin Immunol.

[11] Fadeyi M, Tran T (2013). Calculating the dose of subcutaneous immunoglobulin for primary immunodeficiency disease in patients switched from intravenous to subcutaneous immunoglobulin without the use of a dose-adjustment coefficient. P T.

[12] Kearns S, Kristofek L, Bolgar W, Seidu L, Kile S (2017). Clinical profile, dosing, and quality-of-life outcomes in primary immune deficiency patients treated at home with immunoglobulin G: Data from the IDEaL Patient Registry. J Manag Care Spec Pharm..

[13] Shrestha P, Karmacharya P, Wang Z, Donato A, Joshi AY (2019). Impact of IVIG vs. SCIG on IgG trough level and infection incidence in primary immunodeficiency diseases: A systematic review and meta-analysis of clinical studies. World Allergy Organ J..

[14] Farrugia A, Grazzini G, Quinti I, Candura F, Profili S, Liumbruno GM (2019). The growing importance of achieving national self-sufficiency in immunoglobulin in Italy. The emergence of a national imperative. Blood Transfus..

[15] Picard C, Al-Herz W, Bousfiha A, Casanova JL, Chatila T, Conley ME (2015). Primary immunodeficiency diseases: an update on the classification from the international union of immunological societies expert committee for primary immunodeficiency 2015. J Clin Immunol.

[16] Modell V, Quinn J, Ginsberg G, Gladue R, Orange J, Modell F (2017). Modeling strategy to identify patients with primary immunodeficiency utilizing risk management and outcome measurement. Immunol Res.

[17] Murphy MSQ, Tinmouth A, Goldman M, Chassé M, Colas JA, Saidenberg E (2019). Trends in IVIG use at a tertiary care Canadian center and impact of provincial use mitigation strategies: 10-year retrospective study with interrupted time series analysis. Transfusion.

[18] BC Provincial Blood Coordinating Office. Intravenous Immune Globulin (IVIg) Utilization Management Program Recommendations. 2019 https://www.pbco.ca/images/Programs/IVIG_Provincial_Program/UMIVIG0007_IVIG_Utilization_Management_Program_Guidelines_V42.pdf] (Accessed 05 June 2023).

[19] The Ontario Regional Blood Coordinating Network. Immune Globulin Toolkit for Ontario. 2018 https://transfusionontario.org/wp-content/uploads/2020/05/Immune-globulin-toolkit-for-ontario-v3.0-1.pdf] (Accessed 05 June 2023).

[20] Criteria for the Clinical Use of Immune Globulin-First Edition 2018 https://www.ihe.ca/download/criteria_for_the_clinical_use_of_immune_globulin_first_edition.pdf (Accessed 05 June 2023).

[21] Tricco AC, Lillie E, Zarin W, O'Brien KK, Colquhoun H, Levac D (2018). PRISMA extension for scoping reviews (PRISMA-ScR): checklist and explanation. Ann Intern Med.

[22] Alberta Health Services. Subcutaneous Immunoglobulin Home Infusion Program https://www.albertahealthservices.ca/findhealth/Service.aspx?id=1083521&serviceAtFacilityID=1131984 (Accessed 6 Aug 2023).

[23] Government of Newfoundland and Labrador, Department of Health and Community Services Provincial Blood Coordinating Program. Subcutaneous Immune globulin (SCIG) Home Infusion Program. https://www.gov.nl.ca/hcs/files/bloodservices-pdf-guide-scig-home-infusion.pdf (Accessed 6 Aug 2023).

[24] CSL Behring's Hizentra®CARE program. https://hcp.cslbehring.ca/-/media/product/hcp-portal-ca/documents/hizentra/hizentra-patient-support-program-brochure.pdf (Accessed 6 Aug 2023).

[25] Octapharma’s Cutaquig Patient Support Program. https://www.blood.ca/sites/default/files/CBS_letter-Cutaquig_product_info_ProCare-_Final-01242020-EN.PDF (Accessed 6 Aug 2023).

[26] Jutras C, Robitaille N, Sauthier M, Du Pont-Thibodeau G, Lacroix J, Trottier H (2021). Intravenous Immunoglobulin Use In Critically Ill Children. Clinical Investigat Med Medecine clinique et experimentale..

[27] Hsia CC, Liu Y, Eckert K, Monga N, Elia-Pacitti J, Heddle NM (2015). Intravenous immunoglobulin (IVIg) utilization in immune thrombocytopenia (ITP): a multicenter, retrospective review. Drugs Real World Outcomes.

[28] Liu J, Pavenski K, Sholzberg M (2019). Appropriateness of intravenous immunoglobulin use in immune thrombocytopenia (ITP): a Canadian centre deep dive audit. Transfusion Apheresis Sci.

[29] Tran A, Marcon K, Zamar D, Mi J, Shad J, Zheng J (2023). Evaluation of immunoglobulin replacement therapy in secondary immunodeficiency at three British Columbia hospitals. Vox Sang.

[30] Shabani-Rad M, Zolfaghari S, Hendry J, McCarthy J, Baskin L (2018). 35th International Congress of the ISBT, Toronto, Canada, June 2–6, 2018. Vox Sanguinis..

[31] Bourque PR, Pringle CE, Cameron W, Cowan J, Chardon JW (2016). Subcutaneous immunoglobulin therapy in the chronic management of myasthenia gravis: a retrospective cohort study. PLoS One..

[32] Alcantara M, Sarpong E, Barnett C, Katzberg H, Bril V (2021). Chronic immunoglobulin maintenance therapy in myasthenia gravis. Eur J Neurol.

[33] Suleman A, Theoret L, Bourque P, Pringle E, Cameron DW, Cowan J (2019). Evaluation of a Personalized Subcutaneous Immunoglobulin Treatment Program for Neurological Patients. Canadian J Neurol Sci Le journal canadien des sciences neurologiques.

[34] Gerth WC, Betschel SD, Zbrozek AS (2014). Implications to payers of switch from hospital-based intravenous immunoglobulin to home-based subcutaneous immunoglobulin therapy in patients With primary and secondary immunodeficiencies in Canada. Allergy Asthma Clin Immunol.

[35] Fu LW, Song C, Isaranuwatchai W, Betschel S (2018). Home-based subcutaneous immunoglobulin therapy vs hospital-based intravenous immunoglobulin therapy: a prospective economic analysis. Annals Allergy Asthma Immunol.

[36] Ritchie B, Martins KJB, Tran DT, Blain H, Richer L, Klarenbach SW (2022). Economic impact of self-administered subcutaneous versus clinic-administered intravenous immunoglobulin G therapy in Alberta, Canada: a population-based cohort study. Allergy Asthma Clin Immunol.

[37] Reid B, Pires L (2014). Home gammaglobulin therapy: a patient survey of intravenous and subcutaneous options in Canada. LymphoSign J..

[38] Sholapur NS, Hamilton K, Butler L, Heddle NM, Arnold DM (2016). An evaluation of overall effectiveness and treatment satisfaction with intravenous immunoglobulin among patients with immune thrombocytopenia. Transfusion.

[39] Sultan S, Rondeau É, Levasseur MC, Dicaire R, Decaluwe H, Haddad É (2017). Quality of life, treatment beliefs, and treatment satisfaction in children treated for primary immunodeficiency with SCIg. J Clin Immunol.

[40] Zhou A, Maltez N, Ivory C (2021). ACR convergence 2021 abstract supplement. Arthritis Rheumatol.

[41] Mallick R, Solomon G, Bassett P, Zhang X, Patel P, Lepeshkina O (2022). Immunoglobulin replacement therapy in patients with immunodeficiencies: impact of infusion method on patient-reported outcomes. Allergy Asthma Clin Immunol.

[42] Streu E, Banerji V, Dhaliwal DHS. The efficacy and cost effectiveness of subcutaneous immunoglobulin (SCIG) replacement in patients with immune deficiency secondary to chronic lymphocytic leukemia. Blood Conference: 58th Annual Meeting of the American Society of Hematology, ASH. 2016; 128(22).

[43] Mallon A, Blackmore D, Siddiqi Z. A phase II trial to assess the efficacy, safety and feasibility of 20[percnt] subcutaneous immunoglobulin in patients with myasthenia gravis exacerbation-interim analysis of safety and feasibility. Neurology Conference: 68th American Academy of Neurology Annual Meeting, AAN. 2016; 86(16 SUPPL. 1).

[44] Siddiqi ZA, Beecher G, Anderson D (2018). 15th international congress on neuromuscular diseases, July 6–10, 2018 vienna Austria. J Neuromuscul Dis.

[45] Walter G, Kalicinsky C, Warrington R, Miguel M, Reyes J, Rubin TS (2020). Delivery of subcutaneous immunoglobulin by rapid "push" infusion for primary immunodeficiency patients in Manitoba: a retrospective review. Allergy Asthma Clin Immunol.

[46] Keith PK, Cowan J, Kanani A, Kim H, Lacuesta G, Lee JK (2022). Transitioning subcutaneous immunoglobulin 20% therapies in patients with primary and secondary immunodeficiencies: Canadian real-world study. Allergy Asthma Clin Immunol.

[47] Kobayashi RH, Litzman J, Melamed I, Mandujano JF, Kobayashi AL, Ritchie B (2022). Long-term efficacy, safety, and tolerability of a subcutaneous immunoglobulin 16.5% (cutaquig^®^) in the treatment of patients with primary immunodeficiencies. Clin Exp Immunol..

[48] Brownlee S, Allen C, Kana'an MF, Cameron DW, Cowan J (2022). Cutaquig((R)) is well tolerated in immunodeficient patients who did not tolerate other subcutaneous immunoglobulin products. Hematol Rep.

[49] Abadeh A, Betschel S, Waserman S, Cameron DW, Cowan J (2023). Abstracts poster. Allergy.

[50] Arnold DM, Heddle NM, Cook RJ, Hsia C, Blostein M, Jamula E (2020). Perioperative oral eltrombopag versus intravenous immunoglobulin in patients with immune thrombocytopenia: a non-inferiority, multicentre, randomised trial. Lancet Haematol.

[51] Kaur MN, Arnold DM, Heddle NM, Cook RJ, Hsia C, Blostein M (2022). Cost-effectiveness of eltrombopag vs intravenous immunoglobulin for the perioperative management of immune thrombocytopenia. Blood Adv.

[52] Furlan J, Barth D, Tapia CB, Bril V. Intravenous immunoglobulin versus plasma exchange in the management of patients with myasthenia gravis: A cost-minimization analysis. Neurology Conference: 68th American Academy of Neurology Annual Meeting, AAN. 2016; 86(16 SUPPL. 1).

[53] The National Advisory Committee on Blood and Blood Products (NAC). Providing guidance for Canadians coast to coast to coast. https://nacblood.ca/en (Accessed 5 June 2023).

[54] The National Advisory Committee on Blood and Blood Products (NAC). NAC Statement on Fibrinogen Concentrate Use in Acquired Hypofibrinogenemia. https://nacblood.ca/sites/default/files/2021-10/2021%20FC%20Statement%20Update20210310.pdf (Accessed 5 June 5 2023).

[55] The National Advisory Committee on Blood and Blood Products (NAC). Recommendations for use of Prothrombin Complex Concentrates in Canada. https://nacblood.ca/sites/default/files/2023-02/PCC-Recommendations%20_Revision%20Feb%202022%20_Posted%20April%202022%20-%202023%20Edit.pdf (Accessed 05 June 2023).

[56] Bonilla FA (2008). Intravenous immunoglobulin: adverse reactions and management. J Allergy Clin Immunol.

[57] Ness S (2019). Differentiating characteristics and evaluating intravenous and subcutaneous immunoglobulin. Am J Manag Care.

[58] Skoda-Smith S, Torgerson TR, Ochs HD (2010). Subcutaneous immunoglobulin replacement therapy in the treatment of patients with primary immunodeficiency disease. Ther Clin Risk Manag.

[59] Chow S, Salmasi G, Callum JL, Lin Y (2012). Trimming the fat with an IVIG approval process. Transfusion Apheresis Sci.

