# Supplementary material for: Immunoglobulin utilization in Canada: a comparative analysis of provincial guidelines and a scoping review of the literature
Source: Allergy Asthma Clin Immunol. 2023 Sep 16;19:85. doi: 10.1186/s13223-023-00841-z (PMC10504792; doi:10.1186/s13223-023-00841-z)
Supplement: Supplementary file 1 — Additional file 1: Table S1. Provincial guidelines on the use of IG products in Canada. Table S2. Scoping review search terms. Table S3: The key findings of the selected publications. Table S4. Preferred reporting items for systematic reviews and meta-analyses extension for scoping reviews (PRISMA-ScR) checklist. Table S5. Summary of medical conditions by specialty in provincial guidelines. [file 13223_2023_841_MOESM1_ESM.docx]

**Additional Materials:**

Manuscript title: Immunoglobulin Utilization in Canada: a comparative analysis of provincial guidelines and a scoping review of the Literature

Contents

[**Additional file 1: Table S1**: Provincial guidelines on the use of IG products in Canada. 2](#_Toc143884186)

[**Additional file 1: Table S2:** Scoping review search terms. 5](#_Toc143884187)

[**Additional file 1: Table S3**: The key findings of the selected publications. 6](#_Toc143884188)

[**Additional file 1: Table S4:** Preferred reporting items for systematic reviews and meta-analyses extension for scoping reviews (PRISMA-ScR) checklist. 12](#_Toc143884189)

[**Additional file 1: Table S5:** Summary of medical conditions by specialty in provincial guidelines. 15](#_Toc143884190)

## **Additional file 1: Table S1**: Provincial guidelines on the use of IG products in Canada.

| **Guideline** | **How to Find** | **Link** | **Description** | **How to Access Dose Calculator** | **How to Access Home Infusion Program** |
| --- | --- | --- | --- | --- | --- |
| “*Intravenous Immune Globulin (IVIg) Utilization Management Program Recommendations*”  **Province:** British Columbia  **Year:** 2019  **Version:** V5 | More information can be found on the BC Provincial Blood Coordinating Office webpage:  <https://www.pbco.ca/index.php/blood-products/ivig> | <https://pbco.ca/images/Programs/IVIG_Provincial_Program/UMIVIG0007_IVIG_Utilization_Management_Program_Guidelines_V42.pdf> | This guideline was released in 2019 and is the fifth iteration of the IG utilization guideline for the province of British Columbia. The BC Provincial Blood Coordinating Office is the organizing committee that undertook the creation of this guideline. This guideline includes limited materials relating to recommendation categories and dosing. | <https://pbco.ca/IVIG_Dosing_Calculator.htm> | <https://transfusionontario.org/wp-content/uploads/2020/06/SCIG_patient_handbook.pdf> |
| “*Criteria for the Clinical Use of Immune Globulin*”  **Province:** Alberta, Saskatchewan, Manitoba  **Year:** 2022  **Version:** V2 | More information can be found on the Institute of Health Economics webpage:  <https://www.ihe.ca/research-programs/guideline-adaptation-and-development/immune-globulin-guideline> | <https://www.ihe.ca/public/uploaded/Prairie%20Ig%20Final%20Guideline%2009.02.22.pdf> | This guideline was released in 2022 and is the second iteration of the IG management framework for Alberta, Saskatchewan, and Manitoba provinces. The Inter-Provincial Medical Expert Committee and the Institute of Health Economics are the organizing committees that created this guideline. This guideline includes important materials such as dosing, SCIG administration, follow-up and effectiveness assessment frequency, instructions for weaning patients off IG, off-label use, vaccination, and adverse effects. | <https://www.albertahealthservices.ca/webapps/labservices/IVIG_Dosing_Calculator.htm> | <https://www.albertahealthservices.ca/findhealth/Service.aspx?id=1083521&serviceAtFacilityID=1131984> |
| *“Ontario Immune Globulin (IG) Utilization Management Guidelines”*  **Province:** Ontario  **Year:** 2018  **Version**: V4 | More information can be found on the Ontario Regional Blood Coordinating Network webpage:  <https://transfusionontario.org/en/ontario-ig-utilization-management-guidelines/> | <https://transfusionontario.org/wp-content/uploads/2020/06/ontario-ig-utilization-management-guidelines-v4.0.pdf> | This guideline was released in 2018 and is the fourth iteration of the IG utilization management guideline for the province of Ontario. The Ontario Regional Blood Coordinating Network is the organizing committee that created this guideline. This guideline includes important materials such as IG request forms, updated dose calculator, infusion guide and adverse reaction chart, facts for outpatients, and documents for patients travelling with IG. | <https://ivig.transfusionontario.org/dose> | <https://transfusionontario.org/wp-content/uploads/2020/06/Home-Infusion-Toolkit-v2.0-1.pdf> |
| “*Intravenous Immunoglobulin (IVIg)*”  **Province:** Quebec  **Year:** 2017-2022  **Version:** N/A | More information can be found on the INESSS webpage:  <https://www.inesss.qc.ca/outils-cliniques/outils-cliniques/outils-par-types/guides-dusage-optimal.html> | Neurology:  <https://www.inesss.qc.ca/fileadmin/doc/INESSS/Rapports/Traitement/Guide_Immunoglobulines-EN-WEB.pdf>  Hematology:  <https://www.inesss.qc.ca/fileadmin/doc/INESSS/Rapports/Medicaments/GUIDE_Immunoglobulines-Hematologie-ENG.pdf>  Immunology:  <https://www.inesss.qc.ca/fileadmin/doc/INESSS/Rapports/Medicaments/GUIDE_Immunoglobulines-Immuno-clinique-EN-WEB_VF.pdf>  Rheumatology:  <https://www.inesss.qc.ca/fileadmin/doc/INESSS/Rapports/Medicaments/GUIDE_Immunoglobulines-Rhumato-EN-WEB.pdf>  Dermatology:  <https://www.inesss.qc.ca/fileadmin/doc/INESSS/Rapports/Medicaments/INESSS_OUG_IVIg_Dermato_EN.pdf>  Infectious Diseases:  <https://www.inesss.qc.ca/fileadmin/doc/INESSS/Rapports/Medicaments/GUIDE_Immunoglobulines-infectiologie-ENG-WEB_VF.pdf>  Solid Organ Transplant:  <https://www.inesss.qc.ca/fileadmin/doc/INESSS/Rapports/Usage_optimal/GUIDE_Immunoglobulin-Organ-Transplant.pdf> | This guideline included separate documents for each indication and was released from 2017 to 2022 for the province of Quebec. The Institut National d’Excellence en Sante et en Services Sociaux (INESSS) is the organizing committee that created this guideline. This guideline includes important materials such as dosing, frequency of administration, transfusion reactions, relative contraindications, and main precautions concerning IVIg. | N/A | N/A |
| “*Atlantic Clinical Indications and Criteria for Intravenous and Subcutaneous Immunoglobulin (IVIG/SCIG)*”  **Province:** New Brunswick, Nova Scotia, Prince Edward Island, and Newfoundland and Labrador  **Year:** 2022 **Version:** V2 | More information can be found on the Government of Newfoundland and Labrador webpage:  <https://www.gov.nl.ca/> | <https://src.healthpei.ca/sites/src.healthpei.ca/files/Blood%20Program/Atlantic_Clinical_Indications_and_Criteria_for_Intravenous_and_Subcutaneous_Immunoglobulin.pdf> | This guideline was released in 2022 and is the second iteration of the IG utilization guideline for New Brunswick, Nova Scotia, Prince Edward Island, and Newfoundland and Labrador. The Atlantic Blood Utilization Strategy (ABUS) Working Group is the organizing committee that created this guideline. This guideline includes important materials such as dosing, SCIG administration, and additional indications. | <https://www.nshealth.ca/IVIG/> | <https://www.cdha.nshealth.ca/system/files/sites/documents/atlantic-guidelines-subcutaneous-immune-globulin-home-administration-programs.pdf> |

## **Additional file 1: Table S2:** Scoping review search terms.

| **MEDLINE** | 1. exp Immunoglobulins, Intravenous/ or Immunoglobulins, Intravenous.mp. 2. SCIG.mp. or IVIG.mp. 3. exp Canada/ or Canada.mp. 4. 1 or 2 5. 3 and 4 6. limit 5 to yr="2014 -Current" |
| --- | --- |
| **EMBASE** | 1. exp Immunoglobulins, Intravenous/ or Immunoglobulins, Intravenous.mp. 2. SCIG.mp. 3. IVIG.mp. 4. exp Canada/ or Canada.mp. 5. 1 or 2 or 3 6. 4 and 5 7. limit 6 to yr="2014 -Current" 8. limit 7 to "remove medline records" 9. limit 8 to conference abstracts |
| **PubMed** | ("immunoglobulins, intravenous"[MeSH Terms] OR ("immunoglobulins"[All Fields] AND "intravenous"[All Fields]) OR "intravenous immunoglobulins"[All Fields] OR ("immunoglobulins"[All Fields] AND "intravenous"[All Fields]) OR "immunoglobulins intravenous"[All Fields] OR "SCIG"[All Fields] OR ("immunoglobulins, intravenous"[MeSH Terms] OR ("immunoglobulins"[All Fields] AND "intravenous"[All Fields]) OR "intravenous immunoglobulins"[All Fields] OR "ivig"[All Fields])) AND "canada*"[All Fields] AND 2014/01/01:3000/01/01[Date - Publication] |
| **SCOPUS** | IVIG  TITLE-ABS-KEY ( {intravenous immunoglobulin} ) OR ( {IVIG} ) AND ( PUBYEAR > 2013 ) AND ( LIMIT-TO ( OA , "all" ) ) AND ( LIMIT-TO ( AFFILCOUNTRY , "Canada" ) )  SCIG  TITLE-ABS-KEY ( {subcutaneous immunoglobulin} )  OR  ( {SCIG} )  AND  ( PUBYEAR  >  2013)  AND  (  LIMIT-TO ( OA ,  "all" ) )  AND  ( LIMIT-TO ( AFFILCOUNTRY ,  "Canada" ) ) |

## **Additional file 1: Table S3**: The key findings of the selected publications.

| **Study** | **Key Findings** |
| --- | --- |
| Alcantara et al. (2021) [1] | Patients can successfully transition to IVIG and from IVIG to SCIG in the chronic treatment of generalized MG. |
| Arnold et al. (2020) [2] | Eltrombopag was an effective alternative to intravenous immunoglobulin for the perioperative treatment of ITP. However, the use of eltrombopag might increase the risk of might increase risk of thrombosis. |
| Bourque et al. (2016) [3] | SCIG may be a valuable treatment option for the chronic management of MG. |
| Brownlee et al. (2022) [4] | Cutaquig may be an alternative option for patients who cannot tolerate 20% SCIG products. |
| Fu et al. (2018) [5] | Home-based SCIG therapy was associated with lower costs than hospital-based IVIG therapy. |
| Gerth et al. (2014) [6] | Switching from IVIG to SCIG has the potential to help alleviate nurse shortages and reduce overall healthcare costs in Canada. |
| Hsia et al. (2015) [7] | With the existing practice pattern, the healthcare system's short- and long-term IVIG utilization for ITP will remain very expensive. A constant increase of approximately 2 % per year is predicted for the provincial cost of IVIG use in ITP from 2013 to 2018. |
| Jutras et al. (2021) [8] | 79% of IVIG was administrated for off-label indications. The use of IVIG has increased significantly in the past decade, causing challenges for blood suppliers. |
| Kaur et al. (2022) [9] | In patients with ITP, the use of perioperative eltrombopag was both more effective and cheaper than IVIG. |
| Keith et al. (2022) [10] | The study confirmed the feasibility of infusing Ig20Gly (a concentrated SCIG preparation) either manually or  using a pump after switching from another SCIG product in patients with PID or SID. |
| Kobayashi et al. (2022) [11] | The long-term in-home use of cutaquig (an SCIG preparation) is effective and well tolerated by adult and pediatric PID patients. |
| Liu et al. (2019) [12] | IVIG use was generally appropriate and carefully considered. |
| Mallick et al. (2022) [13] | The SCIG recipients reported better treatment satisfaction than IVIG patients regarding perceived effectiveness. Respondents receiving SCIG reported significantly quicker infusion preparation time, actual infusion time, and post-infusion clean-up time per infusion. Patients who transitioned from IVIG to SCIG were overall satisfied with the experience, with many respondents reporting improved health-related quality of life, productivity, physical and mental health, and greater treatment satisfaction and compliance. |
| Murphy et al. (2019) [14] | The use of IVIG increased over the ten years with an average annual increase of 7.4% but slowed down after the implementation of provincial use mitigation strategies. |
| Reid et al. (2014) [15] | Patients significantly preferred IVIG over SCIG at that time. The loss of time and travel-associated costs with hospital-based programs were patients' concerns regarding the hospital-based programs. |
| Ritchie et al. (2022) [16] | Self-administered SCIG treatment was less costly among adults and children than IVIG. |
| Shih et al. (2017) [17] | The use of the Ontario IVIG Utilization Management Strategy and the completion of the IVIG Request Form were not compliant. |
| Sholapur et al. (2016) [18] | Patients perceived the IVIG treatment as inconvenient but expressed satisfaction with its tolerability for ITP management. |
| Suleman et al. (2019) [19] | A high success rate was reported in transitioning patients from IVIG to SCIG. SCIG treatment is cost-effective compared to IVIG. |
| Sultan et al. (2017) [20] | SCIG treatment is well-received by children. |
| Tran et al. (2023) [21] | 48% of IG replacement therapies in SID patients were deemed inappropriate. More stringent local guidelines and processes for assessing initial and ongoing IG replacement are warranted. |
| Walter et al (2014) [22] | SCIG administration via push in PID patients is an effective IgG replacement method, with most patients preferring to continue this therapy once initiated. |
| Abadeh et al. (2023) [23] | Both IVIG and SCIG treatments were effective in patients with SID by reducing the number of infections and emergency department visits and improving patient perception of health compared to before treatment. |
| Furlan et al. (2016) [24] | Cost-minimizing treatment differs from a public health care insurer's and hospital administrations' perspectives. |
| Mallon et al. (2016) [25] | Despite its large volume SCIG appears to be well tolerated at the standard IVIG dose for neuromuscular diseases. |
| Shabani-Rad et al. (2018) [26] | 85% of IVIG usage cases were labelled as appropriate. Adult neurology, immune deficiency (PID & SID) and hematology (ITP) patients accounted for most registered patients. |
| Siddiqi et al. (2018) [27] | SCIG is effective in the treatment of mild to moderate myasthenia exacerbation. |
| Streu et al. (2016) [28] | The implementation of SCIG resulted in significant cost savings, efficacy, improved quality of life, and treatment satisfaction. |
| Zhou et al. (2021) [29] | While most patients were satisfied with the effectiveness of IVIG treatment, many found it inconvenient mainly due to side effects. However, their willingness to switch to SCIG was also not considerable, possibly due to unfamiliarity with the treatment. |

**References:**

[1] Alcantara M, Sarpong E, Barnett C, Katzberg H, Bril V. Chronic immunoglobulin maintenance therapy in myasthenia gravis. Eur J Neurol. 2021;28(2):639-46. <https://doi.org/10.1111/ene.14547>

[2] Arnold DM, Heddle NM, Cook RJ, Hsia C, Blostein M, Jamula E, et al. Perioperative oral eltrombopag versus intravenous immunoglobulin in patients with immune thrombocytopenia: a non-inferiority, multicentre, randomised trial. The Lancet Haematology. 2020;7(9):e640-e8. <https://doi.org/10.1016/s2352-3026(20)30227-1>

[3] Bourque PR, Pringle CE, Cameron W, Cowan J, Chardon JW. Subcutaneous Immunoglobulin Therapy in the Chronic Management of Myasthenia Gravis: A Retrospective Cohort Study. PLoS One. 2016;11(8):e0159993. <https://doi.org/10.1371/journal.pone.0159993>

[4] Brownlee S, Allen C, Kana'an MF, Cameron DW, Cowan J. Cutaquig((R)) Is Well Tolerated in Immunodeficient Patients Who Did Not Tolerate Other Subcutaneous Immunoglobulin Products. Hematol Rep. 2022;14(4):342-8. <https://doi.org/10.3390/hematolrep14040048>

[5] Fu LW, Song C, Isaranuwatchai W, Betschel S. Home-based subcutaneous immunoglobulin therapy vs hospital-based intravenous immunoglobulin therapy: A prospective economic analysis. Annals of allergy, asthma & immunology : official publication of the American College of Allergy, Asthma, & Immunology. 2018;120(2):195-9. <https://doi.org/10.1016/j.anai.2017.11.002>

[6] Gerth WC, Betschel SD, Zbrozek AS. Implications to payers of switch from hospital-based intravenous immunoglobulin to home-based subcutaneous immunoglobulin therapy in patients with primary and secondary immunodeficiencies in Canada. Allergy, asthma, and clinical immunology : official journal of the Canadian Society of Allergy and Clinical Immunology. 2014;10(1):23. <https://doi.org/10.1186/1710-1492-10-23>

[7] Hsia CC, Liu Y, Eckert K, Monga N, Elia-Pacitti J, Heddle NM. Intravenous Immunoglobulin (IVIg) Utilization in Immune Thrombocytopenia (ITP): A Multi-Center, Retrospective Review. Drugs - real world outcomes. 2015;2(1):35-42. <https://doi.org/10.1007/s40801-015-0009-6>

[8] Jutras C, Robitaille N, Sauthier M, Du Pont-Thibodeau G, Lacroix J, Trottier H, et al. Intravenous Immunoglobulin Use In Critically Ill Children. Clinical and investigative medicine Medecine clinique et experimentale. 2021;44(3):E11-8. <https://doi.org/10.25011/cim.v44i3.36532>

[9] Kaur MN, Arnold DM, Heddle NM, Cook RJ, Hsia C, Blostein M, et al. Cost-effectiveness of eltrombopag vs intravenous immunoglobulin for the perioperative management of immune thrombocytopenia. Blood advances. 2022;6(3):785-92. <https://doi.org/10.1182/bloodadvances.2021005627>

[10] Keith PK, Cowan J, Kanani A, Kim H, Lacuesta G, Lee JK, et al. Transitioning subcutaneous immunoglobulin 20% therapies in patients with primary and secondary immunodeficiencies: Canadian real-world study. Allergy, asthma, and clinical immunology : official journal of the Canadian Society of Allergy and Clinical Immunology. 2022;18(1):70. <https://doi.org/10.1186/s13223-022-00709-8>

[11] Kobayashi RH, Litzman J, Melamed I, Mandujano JF, Kobayashi AL, Ritchie B, et al. Long-term efficacy, safety, and tolerability of a subcutaneous immunoglobulin 16.5% (cutaquig®) in the treatment of patients with primary immunodeficiencies. Clinical and experimental immunology. 2022;210(2):91-103. <https://doi.org/10.1093/cei/uxac092>

[12] Liu J, Pavenski K, Sholzberg M. Appropriateness of intravenous immunoglobulin use in immune thrombocytopenia (ITP): A Canadian centre deep dive audit. Transfusion and apheresis science : official journal of the World Apheresis Association : official journal of the European Society for Haemapheresis. 2019;58(4):491-4. <https://doi.org/10.1016/j.transci.2019.05.003>

[13] Mallick R, Solomon G, Bassett P, Zhang X, Patel P, Lepeshkina O. Immunoglobulin replacement therapy in patients with immunodeficiencies: impact of infusion method on patient-reported outcomes. Allergy, asthma, and clinical immunology : official journal of the Canadian Society of Allergy and Clinical Immunology. 2022;18(1):110. <https://doi.org/10.1186/s13223-022-00746-3>

[14] Murphy MSQ, Tinmouth A, Goldman M, Chassé M, Colas JA, Saidenberg E, et al. Trends in IVIG use at a tertiary care Canadian center and impact of provincial use mitigation strategies: 10-year retrospective study with interrupted time series analysis. Transfusion. 2019;59(6):1988-96. <https://doi.org/10.1111/trf.15271>

[15] Reid B, Pires L. Home gammaglobulin therapy: a patient survey of intravenous and subcutaneous options in Canada. LymphoSign Journal. 2014;01(01):27-37. <https://doi.org/10.14785/lpsn-2014-0001>

[16] Ritchie B, Martins KJB, Tran DT, Blain H, Richer L, Klarenbach SW. Economic impact of self-administered subcutaneous versus clinic-administered intravenous immunoglobulin G therapy in Alberta, Canada: a population-based cohort study. Allergy, Asthma & Clinical Immunology. 2022;18(1):99. <https://doi.org/10.1186/s13223-022-00735-6>

[17] Shih AW, Jamula E, Diep C, Lin Y, Armali C, Heddle NM, et al. Audit of provincial IVIG Request Forms and efficacy documentation in four Ontario tertiary care centres. Transfus Med. 2017;27(2):122-31. <https://doi.org/10.1111/tme.12391>

[18] Sholapur NS, Hamilton K, Butler L, Heddle NM, Arnold DM. An evaluation of overall effectiveness and treatment satisfaction with intravenous immunoglobulin among patients with immune thrombocytopenia. Transfusion. 2016;56(7):1739-44. <https://doi.org/10.1111/trf.13628>

[19] Suleman A, Theoret L, Bourque P, Pringle E, Cameron DW, Cowan J. Evaluation of a Personalized Subcutaneous Immunoglobulin Treatment Program for Neurological Patients. The Canadian journal of neurological sciences Le journal canadien des sciences neurologiques. 2019;46(1):38-43. <https://doi.org/10.1017/cjn.2018.363>

[20] Sultan S, Rondeau É, Levasseur MC, Dicaire R, Decaluwe H, Haddad É. Quality of Life, Treatment Beliefs, and Treatment Satisfaction in Children Treated for Primary Immunodeficiency with SCIg. Journal of clinical immunology. 2017;37(5):496-504. <https://doi.org/10.1007/s10875-017-0409-3>

[21] Tran A, Marcon K, Zamar D, Mi J, Shad J, Zheng J, et al. Evaluation of immunoglobulin replacement therapy in secondary immunodeficiency at three British Columbia hospitals. Vox sanguinis. 2023;118(4):272-80. <https://doi.org/10.1111/vox.13404>

[22] Walter G, Kalicinsky C, Warrington R, Miguel M, Reyes J, Rubin TS. Delivery of subcutaneous immunoglobulin by rapid "push" infusion for primary immunodeficiency patients in Manitoba: a retrospective review. Allergy, asthma, and clinical immunology : official journal of the Canadian Society of Allergy and Clinical Immunology. 2020;16(34):34. <https://doi.org/10.1186/s13223-020-00431-3>

[23] Abadeh A, Betschel S, Waserman S, Cameron DW, Cowan J. Abstracts Poster. Allergy. 2023;78(S111):56-716. <https://doi.org/10.1111/all.15616>

[24] Furlan J, Barth D, Tapia CB, Bril V. Intravenous immunoglobulin versus plasma exchange in the management of patients with myasthenia gravis: A cost-minimization analysis. Neurology Conference: 68th American Academy of Neurology Annual Meeting, AAN. 2016;86(16 SUPPL. 1).

[25] Mallon A, Blackmore D, Siddiqi Z. A phase II trial to assess the efficacy, safety and feasibility of 20[percnt] subcutaneous immunoglobulin in patients with myasthenia gravis exacerbation-interim analysis of safety and feasibility. Neurology Conference: 68th American Academy of Neurology Annual Meeting, AAN. 2016;86(16 SUPPL. 1).

[26] Shabani-Rad M, Zolfaghari S, Hendry J, McCarthy J, Baskin L. 35th International Congress of the ISBT, Toronto, Canada, June 2-6, 2018. Vox sanguinis. 2018;113 Suppl 1(Suppl 1):5-347. <https://doi.org/10.1111/vox.12658>

[27] Siddiqi ZA, Beecher G, Anderson D. 15th International Congress on Neuromuscular Diseases, July 6 - 10, 2018 Vienna, Austria. Journal of Neuromuscular Diseases. 2018;5(s1):S1-S408. <https://doi.org/10.3233/jnd-189001>

[28] Streu E, Banerji V, Dhaliwal DHS. The efficacy and cost effectiveness of subcutaneous immunoglobulin (SCIG) replacement in patients with immune deficiency secondary to chronic lymphocytic leukemia. Blood Conference: 58th Annual Meeting of the American Society of Hematology, ASH. 2016;128(22).

[29] Zhou A, Maltez N, Ivory C. ACR Convergence 2021 Abstract Supplement. Arthritis Rheumatol. 2021;73 Suppl 9:1-4259. <https://doi.org/10.1002/art.41966>

## **Additional file 1: Table S4:** Preferred reporting items for systematic reviews and meta-analyses extension for scoping reviews (PRISMA-ScR) checklist.

| **SECTION** | **ITEM** | **PRISMA-ScR CHECKLIST ITEM** | **REPORTED**  **ON PAGE #** |
| --- | --- | --- | --- |
| **TITLE** | | | |
| Title | 1 | Identify the report as a scoping review. | Title page |
| **ABSTRACT** | | | |
| Structured summary | 2 | Provide a structured summary that includes (as applicable): background, objectives, eligibility criteria, sources of evidence, charting methods, results, and  conclusions that relate to the review questions and objectives. | Structured abstract |
| **INTRODUCTION** | | | |
| Rationale | 3 | Describe the rationale for the review in the context of what is already known. Explain why the review questions/objectives lend themselves to a scoping review approach. | Introduction (paragraphs 1 and 2) |
| Objectives | 4 | Provide an explicit statement of the questions and objectives being addressed with reference to their key elements (e.g., population or participants, concepts, and context) or other relevant key elements used to conceptualize the review questions and/or objectives. | Introduction (paragraph 3) |
| **METHODS** | | | |
| Protocol and registration | 5 | Indicate whether a review protocol exists; state if and where it can be accessed (e.g., a Web address); and if available, provide registration information, including the registration number. | N/A |
| Eligibility criteria | 6 | Specify characteristics of the sources of evidence used as eligibility criteria (e.g., years considered, language, and publication status) and provide a rationale. | Methods, Literature search strategy and selection criteria |
| Information sources* | 7 | Describe all information sources in the search (e.g., databases with dates of coverage and contact with authors to identify additional sources), as well as the date the most recent search was executed. | Methods, Literature search strategy and selection criteria (paragraph 1) |
| Search | 8 | Present the full electronic search strategy for at least 1 database, including any limits used, such that it could be repeated. | Additional file 1: Table S 2 |
| Selection of sources of evidence† | 9 | State the process for selecting sources of evidence (i.e., screening and eligibility) included in the scoping review. |  |
| Data charting process‡ | 10 | Describe the methods of charting data from the included sources of evidence (e.g., calibrated forms or forms that have been tested by the team before their use, and whether data charting was done independently or in duplicate) and any processes for obtaining and confirming data from investigators. | Methods, Literature search strategy and selection criteria (paragraph 3) |
| Data items | 11 | List and define all variables for which data were sought and any assumptions and simplifications made. | Methods, Literature search strategy and selection criteria (paragraph 3) |
| Critical appraisal of individual sources of evidence § | 12 | If done, provide a rationale for conducting a critical appraisal of included sources of evidence; describe the  methods used and how this information was used in any data synthesis (if appropriate). | N/A |
| Synthesis of results | 13 | Describe the methods of handling and summarizing the data that were charted. | Methods, Literature search strategy and selection criteria (paragraph 3) |
| **RESULTS** | | | |
| Selection of sources of evidence | 14 | Give numbers of sources of evidence screened, assessed for eligibility, and included in the review, with  reasons for exclusions at each stage, ideally using a flow diagram. | Figure 2 |
| Characteristics of sources of evidence | 15 | For each source of evidence, present characteristics for which data were charted and provide the citations. | Table 4, Additional file 1: Table S3 |
| Critical appraisal within sources of evidence | 16 | If done, present data on critical appraisal of included sources of evidence (see item 12). | N/A |
| Results of  individual sources of evidence | 17 | For each included source of evidence, present the  relevant data that were charted that relate to the review questions and objectives. | Table 4, Additional file 1: Table S3 |
| Synthesis of results | 18 | Summarize and/or present the charting results as they relate to the review questions and objectives. | Results, Scoping review of current literature |
| **DISCUSSION** | | | |
| Summary of evidence | 19 | Summarize the main results (including an overview of concepts, themes, and types of evidence available), link to the review questions and objectives, and consider the relevance to key groups. | Discussion, paragraph 4 |
| Limitations | 20 | Discuss the limitations of the scoping review process. | Discussion, paragraphs 5 and 6 |
| Conclusions | 21 | Provide a general interpretation of the results with respect to the review questions and objectives, as well as potential implications and/or next steps. | Discussion, paragraph 7 |
| **FUNDING** | | | |
| Funding | 22 | Describe sources of funding for the included sources of evidence, as well as sources of funding for the scoping  review. Describe the role of the funders of the scoping review. | Funding |

JBI = Joanna Briggs Institute; PRISMA-ScR = Preferred Reporting Items for Systematic reviews and Meta-Analyses extension for Scoping Reviews.

* Where *sources of evidence* (see second footnote) are compiled from, such as bibliographic databases, social media platforms, and Web sites.

† A more inclusive/heterogeneous term used to account for the different types of evidence or data sources (e.g., quantitative and/or qualitative research, expert opinion, and policy documents) that may be eligible in a scoping review as opposed to only studies. This is not to be confused with *information sources* (see first footnote).

‡ The frameworks by Arksey and O’Malley (6) and Levac and colleagues (7) and the JBI guidance (4, 5) refer to the process of data extraction in a scoping review as data charting*.*

§ The process of systematically examining research evidence to assess its validity, results, and relevance before using it to inform a decision. This term is used for items 12 and 19 instead of "risk of bias" (which is more applicable to systematic reviews of interventions) to include and acknowledge the various sources of evidence that may be used in a scoping review (e.g., quantitative and/or qualitative research, expert opinion, and policy document).

*From:* Tricco AC, Lillie E, Zarin W, O'Brien KK, Colquhoun H, Levac D, et al. PRISMA Extension for Scoping Reviews (PRISMAScR): Checklist and Explanation. Ann Intern Med. 2018;169:467–473. [doi: 10.7326/M18-0850.](http://annals.org/aim/fullarticle/2700389/prisma-extension-scoping-reviews-prisma-scr-checklist-explanation)

## **Additional file 1: Table S5:** Summary of medical conditions by specialty in provincial guidelines.

| **Medical Condition Category​** | **Number of indications** | | | | |
| --- | --- | --- | --- | --- | --- |
|  | **Ontario** | **Prairie​** | **Atlantic​** | **British Columbia** | **Québec** |
| Dermatology​ | N = 2 | N = 16​ | N = 16​ | N = 1 | N = 15 |
|  | **Recommended:**  Pemphigus vulgaris and variants  **Not recommended for routine use:**  Toxic epidermal necrolysis/Stevens-Johnson Syndrome | **Do:**  Autoimmune blistering diseases  Pyoderma gangrenosum  Scleromyxedema  Toxic epidermal necrolysis/Stevens–Johnson syndrome  **Do not know:**  Atopic dermatitis  Chronic idiopathic urticaria  Drug reaction with eosinophilia and systemic symptoms (DRESS)  Eosinophilic fasciitis  Livedoid vasculopathy  Mast cell activation syndrome  Morphea  Mycoplasma induced rash and mucositis  Necrobiotic xanthogranuloma  Netherton syndrome  Pretibial myxedema  Psoriasis | **Indicated conditions:**  Scleromyxedema – adult  Systemic Vasculitic Syndromes including Polyarteritis Nodosa and Livedoid Vasculopathy – adult  Scleromyxedema – pediatric  Systemic Vasculitic Syndromes including Polyarteritis Nodosa and Livedoid Vasculopathy – pediatric  **Possibly indicated conditions:**  Chronic Idiopathic Urticaria – adult  Chronic Idiopathic Urticaria – pediatric  Dermatomyositis  Necrobiotic Xanthogranuloma – adult  Necrobiotic Xanthogranuloma – pediatric  Pyoderma Gangrenosum – adult  Pyoderma Gangrenosum – pediatric  Severe Forms of Autoimmune Blistering Diseases (Pemphigus vulgaris, Pemphigus foliaceus, Pemphigoid, Cicatricial Pemphigoid, Linear IgA disease, Epidermolysis bullosa acquisita, Pemphigoid gestationis) – adult  Severe Forms of Autoimmune Blistering Diseases (Pemphigus vulgaris, Pemphigus foliaceus, Pemphigoid, Cicatricial Pemphigoid, Linear IgA disease, Epidermolysis bullosa acquisita, Pemphigoid gestationis) – pediatric  Severe Lupus Erythematosus – adult  Severe Lupus Erythematosus – pediatric  Pediatric atopic dermatitis | **Approved/Recommended:**  Pemphigus vulgaris | **Recommended:**  Pemphigus  Group of pemphigoids: mucous membrane pemphigoid, bullous pemphigoid and gestational pemphigoid  **Possible option:**  Dermatomyositis (including the juvenile form)  Scleredema  Scleromyxedema  Necrobiotic xanthogranuloma  Pyoderma gangrenosum  Pretibial myxedema  Livedoid vasculopathy  **Not recommended:**  Stevens-Johnson syndrome/toxic epidermal necrolysis  Dermatitis herpetiformis  Atopic dermatitis/eczema  Urticaria  **Insufficient data:**  Epidermolysis bullosa acquisita  Linear IgA bullous dermatosis |
| Hematology​ | N = 14 | N = 22​ | N = 16​ | N = 5 | N = 23 |
|  | **Recommended:**  Fetal/Neonatal alloimmune thrombocytopenia  Hemolytic disease of the fetus and newborn  Immune thrombocytopenia – adult  Immune thrombocytopenia – pediatric  Post-transfusion purpura  **Not recommended for routine use:**  Acquired hemophilia  Acquired red cell aplasia  Acquired von Willebrand’s disease  Allogenic bone marrow or stem cell transplantation  Autoimmune hemolytic anemia  Autoimmune neutropenia  Hemolytic transfusion reaction in sickle cell disease  Virus associated hemophagocytic syndrome  Hemolytic transfusion reaction | **Do:**  Feto-maternal/neonatal alloimmune thrombocytopenia  Gestational alloimmune liver disease/alloimmune neonatal hemochromatosis  Hemolytic disease of the fetus  Hemolytic disease of the newborn  Heparin-induced thrombocytopenia  Immune thrombocytopenic purpura – adult  Immune thrombocytopenic purpura – pediatric  Neonatal hemochromatosis, prevention  Neonatal thrombocytopenia secondary to maternal autoimmune disorders  Post-transfusion purpura  Pure red cell aplasia  Vaccine induced immune thrombotic thrombocytopenia/vaccine induced prothrombotic immune thrombocytopenia  **Do not do:**  Coagulation factor inhibitors  Hemolytic uremic syndrome  Hemophagocytic lymphohistiocytosis (HLH)  **Do not know:**  Autoimmune hemolytic anemia  Autoimmune neutropenia  Secondary (acquired) HLH  HLH-associated hypogammaglobulinemia with infection  Sickle cell disease, hyperhemolysis syndrome  Thrombotic thrombocytopenic purpura | **Indicated conditions:**  Immune thrombocytopenia (ITP) – adult  Pregnancy associated ITP  Post-transfusion purpura  Fetal alloimmune thrombocytopenia  Post CAR-T cell therapy  Neonatal alloimmune thrombocytopenia  Hemolytic disease of the newborn  Immune thrombocytopenia – pediatric  Neonates of mothers with ITP  **Possibly indicated conditions:**  Acquired hemophilia with Factor VIII Inhibitor  Factor VIII Inhibitor  Secondary immunodeficiency - adult  Warm autoimmune hemolytic anemia  Hemophagocytic Lymphohistiocytosis  Hematological malignancy  Secondary immunodeficiency – pediatric | **Approved/Recommended:**  Fetal-neonatal alloimmune thrombocytopenia  Hemolytic disease of the newborn  Idiopathic thrombocytopenic purpura – pediatric  Idiopathic thrombocytopenic purpura – adult  **Not recommended:**  Aplastic anemia | **Recommended:**  Allogeneic hematopoietic stem cell transplant (for infection prevention)  Hypogammaglobulinemia secondary to a hematologic cancer  Acute immune thrombocytopenia  Immune thrombocytopenia during pregnancy  Fetal or neonatal alloimmune thrombocytopenia  **Possible option:**  Autoimmune hemolytic anemia  Hemolytic disease of the newborn or fetus  Autoimmune neutropenia  Post-transfusion purpura  Hyperhemolytic syndrome  Catastrophic antiphospholipid syndrome  Evans syndrome  Chronic immune thrombocytopenia  Infection-induced immune thrombocytopenia (HIV, HCV)  **Not recommended:**  Aplastic anemia  Pure red blood cell aplasia  Thrombotic thrombocytopenic purpura  Hemolytic transfusion reaction (without hyperhemolysis)  Hemolytic-uremic syndrome  Secondary hemophagocytic syndrome  Heparin-induced thrombocytopenia  **Insufficient data:**  Parvovirus B19-induced red blood cell aplasia  Acquired hemophilia |
| Immunology​ | N = 2 | N = 2​ | N = 5​ | N = 2 | N = 42 |
|  | **Recommended:**  Primary Immune Deficiency and Secondary Immune Deficiency  Hematopoietic Stem Cell Transplant in primary immunodeficiencies | **Do:**  Hypogammaglobulinemia, secondary  Primary immunodeficiency (PID) disorders | **Indicated conditions:**  Primary Immunodeficiency - adult  Secondary Immunodeficiency - adult  Primary Immunodeficiency - pediatric  Secondary Immunodeficiency - pediatric  **Possibly indicated conditions:**  Chronic Idiopathic Urticaria | **Approved/Recommended:**  Primary immune deficiency  Secondary immune deficiency | **Recommended:**  Severe combined immunodeficiencies  Combined immunodeficiencies  Combined immunodeficiencies with associated or syndromic features  Agammaglobulinemia  Common variable immunodeficiency  Hyper IgM syndrome  Primary isolated IgG deficiency  LRBA or CTLA4 deficiency  WHIM syndrome  Good syndrome  Hypogammaglobulinemia due to CAR-T cell therapies  **Possible option:**  X-linked lymphoproliferative syndromes and other immunodeficiencies associated with susceptibility to Epstein-Barr virus  PLCg2 associated antibody deficiency and immune dysregulation or ADA2 deficiency  Transient hypogammaglobulinemia in infancy  IgG2 subclass deficiency associated or not with IgA deficiency  Specific antibody deficiencies with normal Ig levels  CARD11 gain-of-function mutations  STAT3 gain-of-function mutations  Hypogammaglobulinemia due to treatment with B cell-targeted therapy, including rituximab, or with immunosuppressants, including high-dose corticosteroids, or with a drug  Hypogammaglobulinemia due to Steinert myotonic dystrophy  Hypogammaglobulinemia due to chylothorax, or exudative enteropathy, or intestinal lymphangiectasia, or lymphedema, or nephrotic syndrome  In preterm newborns in an infection treatment context  **Not recommended:**  Selective IgA deficiency with a confirmed diagnosis  Kappa chain deficiency  Primary hemophagocytic lymphohistiocytosis  Autoimmune lymphoproliferative syndromes  Congenital defects in phagocyte number or function  All defects in intrinsic or innate immunity (with the exception of WHIM syndrome)  Auto-inflammatory disorders not associated with an antibody deficiency  All complement deficiencies (except those associated with a syndrome similar to systemic lupus erythematosus)  Phenocopies of primary immunodeficiencies associated with a somatic mutation  Selective IgM deficiency with a confirmed diagnosis  In preterm newborns in an infection prevention context  **Insufficient data:**  Regulatory T cell defects associated with immune dysregulation polyendocrinopathy enteropathy X-linked or CD25 deficiency or BACH2 deficiency  Autoimmunity with or without lymphoproliferation  Immune dysregulation with colitis  Complement deficiencies associated with a syndrome similar to systemic lupus erythematosus  Phenocopies of primary immunodeficiencies associated with auto-antibodies  IgG subclass deficiencies associated or not with IgA deficiency (with the exception of IgG2 subclass deficiency)  Hypogammaglobulinemia due to treatment with plasma cell targeting agents  Secondary hypogammaglobulinemia in a severe burn patient  Hypogammaglobulinemia associated with malnutrition |
| Infectious Disease​ | N = 2 | N = 11​ | N = 4​ | N = 3 | N = 13 |
|  | **Recommended:**  Staphylococcal toxic shock  Invasive Group A streptococcal fasciitis with associated toxic shock | **Do:**  Hepatitis A, post-exposure prophylaxis  Measles, post-exposure prophylaxis (PEP)  Toxic shock syndrome  When VZV immune globulin is unavailable  **Do not do:**  Clostridium difficile infection, recurrent  HIV/AIDS  PEP Immunocompetent individuals older than 12 months  Sepsis, prophylaxis  Severe acute respiratory syndrome coronavirus 2 (SARS-CoV-2)/ COVID-19  Varicella-zoster virus (VZV), prophylaxis  **Do not know:**  Necrotizing fasciitis | **Indicated conditions:**  Group A Streptococcus (GAS) Necrotizing Fasciitis or Toxic Shock Syndrome  Staphylococcus Aureus Toxic Shock Syndrome (TSS)  **Possibly indicated conditions:**  Chronic Parvovirus Infection with Anemia  Measles Post Exposure Prophylaxis | **Approved/Recommended:**  Staphylococcal toxic shock  Invasive Group A streptococcal fasciitis with associated toxic shock  Measles post-exposure prophylaxis | **Possible option:**  Pediatric viral cardiomyopathy  Toxic shock syndrome  Multisystem inflammatory syndrome in children temporally associated with COVID-19  **Not recommended:**  Adult viral cardiomyopathy  COVID-19 (without multisystem inflammatory syndrome)  Clostridioides difficile enterocolitis  Sepsis (except neonatal enterovirus sepsis)  Mycoplasma pneumoniae-induced rash and mucositis  Adult necrotizing fasciitis (without shock criteria)  Infection prevention post trauma or surgery  **Insufficient data:**  Pediatric necrotizing fasciitis  Neonatal enterovirus sepsis  Multisystem inflammatory syndrome in adults temporally associated with COVID-19 |
| Transplant Medicine​ | N = 4 | N = 21​ | N = 3​ | N = 0 | N = 11 |
|  | **Recommended:**  Kidney transplant from living donor to whom the patient is sensitized  Pre-Transplant (heart)  Peri-Transplant (heart, lung, kidney, pancreas)  Post-Transplant | **Do:**  Community-acquired respiratory virus, upper respiratory tract infection (Proven respiratory syncytial virus (RSV) in high-risk patients)  Hematopoietic stem cell transplant (HSCT), allogeneic, Cytomegalovirus (CMV)-induced pneumonitis  Hematopoietic stem cell transplant (HSCT) for primary immunodeficiency (PID) disorders  Parvovirus B19 in solid organ transplant recipients  Solid organ transplant, active antibody-mediated rejection (ABMR) prevention and management  Solid organ transplant, ongoing desensitization, prevention or treatment of graft rejection  **Do not do:**  Community-acquired respiratory virus, upper respiratory tract infection (all other patient groups, including solid organ transplant recipients (other than lung))  Community-acquired respiratory virus, lower respiratory tract infection (all other patient groups, including solid organ transplant recipients (other than lung))  Cytomegalovirus (CMV) infection, prevention  Epstein-Barr virus (EBV)-associated post-transplant lymphoproliferative disorders (PTLD)  Gastrointestinal viruses in solid organ transplant (Refractory and persistent viral gastroenteritis syndromes (other than Norovirus or Rotavirus))  Hematopoietic stem cell transplant (HSCT), allogeneic, graft-versus host disease  Hematopoietic stem cell transplant (HSCT), autologous  Parvovirus B19 in solid organ transplant recipients – prevention  Pulmonary graft-versus-host disease  **Do not know:**  Adenovirus in solid organ transplant recipients  BK polyomavirus nephropathy in solid organ transplant recipients  Non-RSV in high-risk patients  Community-acquired respiratory virus, lower respiratory tract infection (High-risk patients)  Gastrointestinal viruses in solid organ transplant (Refractory and persistent Norovirus or Rotavirus diarrhea)  Kidney transplant, isolated acute/active T-cell mediated rejection (TCMR) management | **Indicated conditions:**  Acute Antibody Mediated Rejection  **Possibly indicated conditions:**  Chronic Parvovirus Infection with Anemia  BK Polyomavirus |  | **Possible option:**  Prevention of transplant rejection in a kidney, heart, lung, pancreas, or small intestine transplant recipient  Prevention of transplant rejection in a liver transplant recipient  Treatment of transplant rejection in a solid organ transplant recipient  Treatment of a parvovirus B19 infection in a solid organ transplant recipient  Treatment of a polyomavirus BK infection in a solid organ transplant recipient  **Not recommended:**  Prevention of the following infections in a solid organ transplant recipient: Epstein-Barr virus, Norovirus, HHV-6, Parvovirus B19, Polyomavirus BK, Adenovirus, West Nile virus  Treatment of the following infections in a solid organ transplant recipient: Epstein-Barr virus, Norovirus, HHV-6  **Insufficient data:**  Prevention of transplant rejection in a hyperimmunized individual who has received a liver transplant  Prevention of a respiratory syncytial virus infection in a solid organ transplant recipient  Treatment of chronic humoral transplant rejection in a solid organ transplant recipient  Treatment of an infection in a solid organ transplant recipient by: the respiratory syncytial virus, an adenovirus, the West Nile virus |
| Neurology | N = 10 | N = 49​ | N = 19​ | N = 12 | N = 27 |
|  | **Recommended:**  Chronic inflammatory demyelinating polyneuropathy  Guillain-Barre syndrome including Miller-Fisher syndrome and variants  Multifocal motor neuropathy  Myasthenia gravis  **Not recommended for routine use:**  Acute disseminated encephalomyelitis  Lambert-Eaton Myasthenic syndrome  Pediatric autoimmune neuropsychiatric disorders associated with streptococcal infections  Rasmussen’s encephalitis  Stiff person’s syndrome  n-methyl-D-aspartate encephalitis | **Do:**  Acute disseminated encephalomyelitis  Autoimmune encephalitis mediated by antibodies (AMAE) targeting cell-surface antigens  Chronic inflammatory demyelinating polyneuropathy  Guillain–Barré syndrome  Lambert–Eaton myasthenic syndrome  Multifocal motor neuropathy  Multiple sclerosis (MS)  Myasthenia gravis (MG)  Myelin oligodendrocyte glycoprotein antibody-associated disorders (MOGAD) – pediatric  Neuropathy associated with IgM paraproteinemia  Opsoclonus-myoclonus ataxia (OMA) – pediatric onset  Sjögren syndrome associated neuropathy  Stiff person syndrome (Moersch–Woltman syndrome)  Sydenham chorea  Vasculitic neuropathy as part of a systemic disorder (systemic vasculitis affecting the peripheral nervous system)  **Do not do:**  Acute optic neuritis  Adrenoleukodystrophy  Alzheimer disease  Autism  Chronic fatigue syndrome (myalgic encephalomyelitis)  Critical illness polyneuropathy  Motor neuron disease  RRMS, long-term therapy  Primary progressive MS; progressive phase of MS without relapse  MG, mild generalized – adult  MG – ocular  Narcolepsy/cataplexy  Neuropathic pain  Axonal neuropathy associated with IgM paraproteinemia  Polyneuropathy, organomegaly, endocrinopathy, monoclonal protein, skin changes (POEMS) syndrome  Post-polio syndrome  **Do not know:**  Acute flaccid myelitis  Aicardi-Goutières syndrome  Childhood epilepsy, drug resistant  Diabetic amyotrophy  Hashimoto encephalopathy  MG – juvenile  Neuromyelitis optica spectrum disorders (NMOSD)  Demyelinating neuropathy with anti-MAG antibodies  Opsoclonus-myoclonus ataxia (OMA) – adult onset  Paraneoplastic neurological syndromes  Pediatric autoimmune neuropsychiatric disorder associated with streptococcal infection (PANDAS)  Postural orthostatic tachycardia syndrome (POTS)  Rasmussen syndrome  Sensory ganglionopathy  Susac syndrome  Sydenham chorea (long-term therapy)  Transverse myelitis  Vasculitic neuropathy, non-systemic (vasculitis solely affecting the peripheral nervous system; isolated vasculitic neuropathy) | **Indicated conditions:**  Chronic inflammatory demyelinating polyneuropathy  Guillain-Barre syndrome - adult  Multifocal motor neuropathy  Myasthenia gravis - adult  Guillain-Barre syndrome – pediatric  Myasthenia Gravis – pediatric  **Possibly indicated conditions:**  Autoimmune encephalitis: N-Methyl-D-Aspartate - adult  Autoimmune encephalitis: Rasmussen’s encephalitis - adult  Autoimmune optic neuropathy  Lambert-Easton Myasthenic syndrome  Multiple sclerosis relapsing/remitting only  Neuromyelitis optica  Anti-myelin aligodendrocyte glycoprotein syndromes  Paraneoplastic cerebellar degeneration  Stiff person syndrome  Acute disseminated encephalomyelitis  Autoimmune encephalitis: N-Methyl-D-Aspartate – pediatric  Autoimmune encephalitis: Rasmussen’s encephalitis - pediatric  Post-streptococcal autoimmune disorders: Pediatric Autoimmune Neuropsychiatric Disorders Associated with Streptococcal Infections (PANDAS), Pediatric Acute onset Neuropsychiatric Syndrome (PANS) and Sydenham’s Chorea | **Approved/Recommended:**  Guillain-Barre syndrome, including Miller-Fisher syndrome and other variants  Chronic inflammatory demyelinating polyneuropathy  Multifocal motor neuropathy  Myasthenia gravis  **Not recommended:**  Adrenoleukodystrophy  Amyotropic lateral sclerosis  Autism  Critical illness polyneuropathy  Inclusion body myositis  Intractable childhood epilepsy  Paraproteinemic neuropathy (IgM variant)  POEMS (polyneuropathy, organomegaly, endocrinopathy, monoclonal gammopathy and skin changes) | **Recommended:**  Myasthenia gravis  Multifocal motor neuropathy  Chronic inflammatory demyelinating polyneuropathy  Guillain-Barré syndrome (or its variants, such as Miller-Fisher syndrome)  **Possible option:**  Dermatomyositis (including the juvenile form)  Acute disseminated encephalomyelitis (ADEM)  Rasmussen’s encephalitis  Myasthenia gravis  Polymyositis (including immune-mediated necrotizing myopathies)  Remitting multiple sclerosis  Stiff person syndrome  Lambert-Eaton myasthenic syndrome  Opsomyoclonus syndrome  **Not recommended:**  Adrenoleukodystrophy  Alzheimer’s disease  Amyotrophic lateral sclerosis  Autism spectrum disorder  IgM paraproteinemic neuropathy  Inclusion body myositis  Critical illness polyneuropathy  Polyneuropathy, organomegaly, endocrinopathy, monoclonal gammopathy, skin changes (POEMS) syndrome  Primary- or secondary-progressive multiple sclerosis  **Insufficient data:**  Autoimmune encephalitis  Diabetic amyotrophy  Neuromyelitis optica  PANDAS (pediatric autoimmune neuropsychiatric disorders associated with streptococcal infections)  Paraneoplastic neuropathy |
| Rheumatology  ​ | N = 3 | N = 22​ | N = 11​ | N = 2 | N = 34 |
|  | **Recommended:**  Juvenile idiopathic inflammatory myopathy (previously Juvenile dermatomyositis)  Kawasaki disease  Idiopathic inflammatory myopathy – includes Dermatomyositis and Polymyositis (does not include Inclusion Body Myositis) | **Do:**  Antiphospholipid syndrome, catastrophic  Autoimmune retinopathy (AIR)  Dermatomyositis – pediatric  Eosinophilic granulomatosis with polyangiitis (EGPA) (Churg–Strauss disease)  Kawasaki disease – pediatric  Macrophage activation syndrome (MAS)  Multisystem inflammatory syndrome in children (MIS-C) associated with SARS-CoV-2/COVID-19 infection  Myopathies, inflammatory – adult: dermatomyositis, polymyositis, necrotizing autoimmune myopathy  **Do not do:**  Antiphospholipid syndrome (other than catastrophic)  Behçet disease  Inclusion body myositis (IBM)  Rheumatoid arthritis  **Do not know:**  Congenital heart block, autoimmune (neonatal lupus)  Immune-mediated uveitis  Kawasaki disease – adult  Multisystem inflammatory syndrome in adults (MIS-A) associated with SARS-CoV-2/COVID-19 infection  Polymyositis – pediatric  Scleroderma  Sjögren syndrome  Systemic juvenile idiopathic arthritis (JIA) and adult Still disease  Systemic lupus erythematosus (SLE)  Vasculitic syndromes | **Indicated conditions:**  Immune-Mediated Inflammatory Myositis  Juvenile Dermatomyositis  Kawasaki Syndrome  Systemic Onset Juvenile Idiopathic Arthritis  **Possibly indicated conditions:**  Catastrophic Antiphospholipid Antibody Syndrome  Adult-onset Still’s Disease  Sjogren’s Syndrome  Hemophagocytic Lymphohistiocytosis – adult  Multisystem Inflammatory Syndrome in Adults  Multisystem Inflammatory Syndrome in Children  Hemophagocytic Lymphohistiocytosis /Macrophage Activation Syndrome – pediatric | **Approved/Recommended:**  Juvenile dermatomyositis  Kawasaki disease | **Recommended:**  Kawasaki disease  **Possible option:**  Juvenile idiopathic arthritis  Adult Still’s disease  Dermatomyositis (including the juvenile form)  Polymyositis (including immune- mediated necrotizing myopathies)  Diffuse or localized systemic sclerosis  Sjögren’s syndrome  Systemic lupus erythematosus  Eosinophilic granulomatosis with polyangiitis (Churg-Strauss syndrome)  Granulomatosis with polyangiitis (Wegener’s syndrome)  Microscopic polyangiitis  Catastrophic antiphospholipid syndrome  Neonatal lupus  **Not recommended:**  Rheumatoid arthritis  Cryopyrinopathies  Inclusion body myositis  Cutaneous lupus  Localized scleroderma (morphea)  Antiphospholipid syndrome (non-catastrophic)  Temporal arteritis  Behçet’s disease  IgA vasculitis  Leukocytoclastic vasculitis  Lymphocytic vasculitis  **Insufficient data:**  Overlap syndromes  Takayasu’s arteritis  Cogan syndrome  Susac syndrome  Primary central nervous system vasculitis  Levamisole vasculopathy  Eosinophilic fasciitis  Sneddon syndrome  Polyarteritis nodosa  Hypocomplementemic urticarial vasculitis syndrome |
